# Controlling Endemic Cholera with Oral Vaccines

**DOI:** 10.1371/journal.pmed.0040336

**Published:** 2007-11-27

**Authors:** Ira M Longini, Azhar Nizam, Mohammad Ali, Mohammad Yunus, Neeta Shenvi, John D Clemens

**Affiliations:** 1 Vaccine and Infectious Disease Institute, Fred Hutchinson Cancer Research Center, Seattle, Washington, United States of America; 2 Department of Biostatistics, School of Public Health and Community Medicine, University of Washington, Seattle, Washington, United States of America; 3 Department of Biostatistics, The Rollins School of Public Health, Emory University, Atlanta, Georgia, United States of America; 4 International Vaccine Institute, Seoul, Korea; 5 International Centre for Diarrhoeal Disease Research, Bangladesh, Dhaka, Bangladesh; Joint Malaria Programme, Tanzania

## Abstract

**Background:**

Although advances in rehydration therapy have made cholera a treatable disease with low case-fatality in settings with appropriate medical care, cholera continues to impose considerable mortality in the world's most impoverished populations. Internationally licensed, killed whole-cell based oral cholera vaccines (OCVs) have been available for over a decade, but have not been used for the control of cholera. Recently, these vaccines were shown to confer significant levels of herd protection, suggesting that the protective potential of these vaccines has been underestimated and that these vaccines may be highly effective in cholera control when deployed in mass immunization programs. We used a large-scale stochastic simulation model to investigate the possibility of controlling endemic cholera with OCVs.

**Methods and Findings:**

We construct a large-scale, stochastic cholera transmission model of Matlab, Bangladesh. We find that cholera transmission could be controlled in endemic areas with 50% coverage with OCVs. At this level of coverage, the model predicts that there would be an 89% (95% confidence interval [CI] 72%–98%) reduction in cholera cases among the unvaccinated, and a 93% (95% CI 82%–99%) reduction overall in the entire population. Even a more modest coverage of 30% would result in a 76% (95% CI 44%–95%) reduction in cholera incidence for the population area covered. For populations that have less natural immunity than the population of Matlab, 70% coverage would probably be necessary for cholera control, i.e., an annual incidence rate of ≤ 1 case per 1,000 people in the population.

**Conclusions:**

Endemic cholera could be reduced to an annual incidence rate of ≤ 1 case per 1,000 people in endemic areas with biennial vaccination with OCVs if coverage could reach 50%–70% depending on the level of prior immunity in the population. These vaccination efforts could be targeted with careful use of ecological data.

## Abstract 2

Using data from Bangladesh, Ira Longini and colleagues develop a mathematical model predicting that oral vaccination of 50%-70% of the population could control cholera transmission in an endemic region.

## Editors' Summary

**Background.:**

Throughout history, there have been devastating outbreaks of cholera—a gut infection characterized by diarrhea and severe dehydration—around the world. These days, cholera is mainly confined to developing countries where it disrupts social structures, impedes economic development, and probably causes about 100,000 deaths a year. People get cholera, which is caused by a bacterium called Vibrio cholerae, by eating food or drinking water contaminated with feces (stools) from an infected person. Most infected people have no or mild symptoms but shed the bug in their feces for up to two weeks. Other people develop severe diarrhea, producing stools that look like water with flecks of rice in it. If untreated, patients with severe cholera can die from dehydration within hours of developing symptoms. The standard treatment for cholera is replacement of the fluids and salts lost through diarrhea by drinking an oral rehydration solution or, in the worst cases, by fluid replacement directly into a vein. Without this treatment, which is not always available in the developing countries where cholera is endemic (always present), one in every two people with severe symptoms die.

**Why Was This Study Done?:**

The best way to control cholera is to ensure that everyone has access to safe water and good sanitation, but this is not possible in some poor countries, in refugee camps, or after natural disasters such as floods. Oral cholera vaccines (preparations given by mouth that stimulate the immune system to attack V. cholerae) are available but they are not 100% effective and the protection they provide wanes over time. Consequently, vaccination has not been adopted as a control measure for endemic cholera. Recently, however, researchers have suggested that oral cholera vaccines induce “herd immunity.” With a disease that passes between people, when most of the population is immune to it, it is unlikely that an infected person will come into contact with a susceptible person and pass the disease on. In effect, both vaccinated and unvaccinated people are protected from the disease. If cholera vaccines do induce herd immunity, then mass immunization might help to control endemic cholera. In this study, the researchers have used a mathematical model to investigate this possibility.

**What Did the Researchers Do and Find?:**

The researchers built a large-scale model of cholera transmission using information about the population of Matlab, Bangladesh (a region where cholera is endemic), together with data on the biology of cholera and data from a large oral vaccine trial done in Matlab in the 1980s. They used this model to predict whether cholera would be controlled after vaccination of different proportions of the population. They found that cholera transmission would be controlled if half the population in the region was vaccinated. This level of vaccine coverage reduced the number of cholera cases among unvaccinated people by 89% and among the entire population by 93%. With only one-third of the population vaccinated, the number of cases of cholera still fell by three-fourths. The model also predicted that in areas where there is less natural immunity to cholera (the people in Matlab are constantly exposed to V. cholerae, so they have some immunity to the bug even without vaccination), 70% of the population would probably need to be vaccinated to control cholera.

**What Do These Findings Mean?:**

These findings suggest that, because of herd immunity, vaccinating only half the population could control cholera transmission in endemic regions where there is a high level of natural immunity. Where there is less natural immunity, more of the population would need to be immunized. Although mass immunization of even 70% of a population should be achievable, for maximal protection against V. cholerae, two doses of the oral cholera vaccine need to be given a week apart followed by a booster every two years. In developing countries this regimen might not always be logistically feasible or affordable. Furthermore, because the accuracy of the model's predictions depends on the assumptions made to construct it and on the data incorporated into it, these findings need to be checked in field trials in other endemic areas. Nevertheless, these findings suggest that public-health officials should consider including mass vaccination in their efforts to control endemic cholera.

**Additional Information.:**

Please access these Web sites via the online version of this summary at http://dx.doi.org/10.1371/journal.pmed.0040336.

- The MedlinePlus encyclopedia contains a page on cholera (in English and Spanish)
- Information is available from the World Health Organization on cholera, including a fact sheet on the disease (in English, Spanish, French, Russian, Arabic, and Chinese)
- The US Centers for Disease Control and Prevention provide information on cholera (in English, Spanish and Portuguese).
- The UK National Health service provides simple information on vaccines and immunization, which includes an animation that explains herd immunity

## Introduction

The global burden of cholera remains substantial. In 2005, 131,943 cases and 2,272 deaths were reported to the WHO, and recently major, sustained epidemics have been reported in West Africa [1]. These statistics are gross underestimates, as many cholera-endemic countries do not report cholera to the WHO, including Bangladesh, which has among the highest rates of cholera in the world. More realistic estimates of the global burden of cholera mortality place the figure at 100,000–150,000 deaths per year. This high burden occurs because cholera targets the most impoverished populations, which often lack access to centers that can appropriately administer life-saving rehydration therapy.

This continuing high burden highlights the need for interventions to prevent cholera. While improved water and sanitation constitute the ultimate basis for the prevention of cholera, this is a far-off goal for the impoverished settings in which cholera thrives. Vaccines constitute near-term options for cholera control. During the past 20 y, killed oral cholera vaccines (OCVs) have been shown to be safe and protective in populations with endemic cholera [2,3]. A large-scale community OCV vaccine trial in Vietnam showed that mass vaccination for endemic cholera is operationally feasible in a developing country setting [4]. Yet, because these vaccines have moderate levels of protective efficacy, they have not been routinely adopted as control measures for endemic cholera [5,6]. Recently, however, a reanalysis of a field trial of killed OCVs in Bangladesh demonstrated that these vaccines are capable of inducing herd protection of nonvaccinees, as well as enhanced protection of vaccinees, when even modest levels of vaccine coverage of the targeted population are attained [7]. These observations prompted us to re-examine the potential impact of use of these vaccines in mass immunization programs on the control of endemic cholera. In this paper, we use a large-scale stochastic simulation model to investigate potential control of endemic cholera with OCV.

## Methods

In the mid-1980s, a randomized controlled vaccine trial with OCV in Matlab, Bangladesh, yielded 70% direct vaccine efficacy for up to two years [2,3]. We use information about Matlab, Bangladesh to construct a model of the population as it was in 1985, consisting of 183,826 participants. These individuals were mapped into families and families were distributed in “baris,” i.e., patrilineally related household clusters [7]. In the model, baris are further clustered into subregions of about 6 km^2^ that are considered to be the geographic cholera transmission areas. The model (see Figures S1–S5 and Texts S1 and S2) represents the number of contacts that a typical person makes with sources of potential cholera transmission in the course of a day. The age and bari size distributions of the population are based on data from Ali et al. [7]. Women and children are assumed to come into contact with sources of infection in the subregion where they live, while working males are assumed to make contact with infective sources in the subregion where they live as well as where they work. The population structure and movement distance function are given in Text S1.

**Figure 1:**
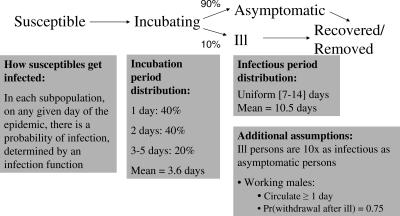
Basic Model Parameter Distributions Modeled natural history of cholera [8–10]. People start out partially susceptible. Newly infected people pass through the incubating state (mean 3.6 d) and infectious state (mean 10.5 d) after which they recover with partial immunity or die. The probability distributions of the incubation and infectious periods are shown in the figure. We assume that 10% of infected people develop overt cholera symptoms and 90% will be asymptomatic. We further assume that symptomatic people are ten times as infectious as asymptomatics. Additionally, this model allows for 75% of symptomatic working males to withdraw to their subregion [Pr(withdrawal after ill)].

The modeled natural history of cholera is described in Figure 1. The distributions of the duration of the incubation and infectious periods and other important natural history parameters are taken from the cholera literature [8–10]. The model was calibrated to cholera illness incidence data from a large cholera vaccine trial in the Matlab field area of the International Centre for Diarrhoeal Disease Research, Bangladesh (ICDDR,B), that took place from 1985 to 1989 [2,3,11]. OCV or placebo (killed E. coli) was offered to children 2–15 y of age and women over 15 y. Matlab has cholera transmission year around, but it generally experiences a large cholera epidemic from September through December and then a somewhat smaller epidemic from March through May [12]. Although the definition of endemic cholera may vary, we define this pattern of cholera transmission to be “endemic,” i.e., at least a low level of cholera all year round, with periodic larger epidemics. For our analysis we assess cholera risk in individuals residing in different subregions in the field trial area. Subregions are useful for cluster analysis because they are geographically discrete with local sources of water. Infected people within subregions can possibly transmit the disease person-to-person [13]; more commonly transmission occurs when feces enter local ponds where vibrios from the feces are amplified in organic material, especially plankton, during the epidemic periods [14]. We define an infection function (see Text S2) that gives each susceptible person's daily probability of infection from all possible sources of infection created by infected people excreting cholera vibrios into the environment or through more direct contact. This probability of infection is proportional to the number of vaccinated and unvaccinated people in the subregion where contact is specified to occur (see Text S2)*.*

**Figure 2:**
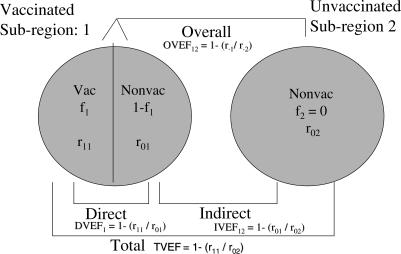
Schematic of Effectiveness Comparisons for Two Subregions Subregion 1 has a fraction *f*_1_ > 0 people vaccinated, while the comparison subregion 2 has nobody vaccinated, i.e., *f*_2_ = 0. We let r*_ij_* be the cholera infection rate for people in subregion *j* with vaccination status *i*, where *i* = 0 for unvaccinated and *i* = 1 for vaccinated. The indirect effect of vaccination is measured by comparing the infection rates between the unvaccinated in the two subregions. Thus, the indirect vaccine effectiveness, i.e., IVEF, when comparing subregion 1 to 2 is IVEF_12_ = 1 − (*r*_01_/*r*_02_). The overall effect of vaccination is measured by comparing the average (over the vaccinated and unvaccinated groups) infection rates between the two subregions. Thus, the overall vaccine effectiveness, i.e., OVEF, is OVEF_12_ = 1 − (*r.*_1_/*r.*_2_), where the · indicates averaging over the vaccinated and unvaccinated. The total effect of vaccination is measured by comparing the infection rate in the vaccinated in subregion 1 to the unvaccinated in subregion 2. Thus, the total vaccine effectiveness, i.e., TVEF, is TVEF_12_ = 1 − (*r*_11_/*r*_02_). The direct effect of vaccination is measured by comparing the infection rates in the vaccinated and unvaccinated in the same subregion. The direct vaccine effectiveness, i.e., DVEF, is DVEF_1_ = 1 − (*r*_11_/*r*_01_). In general, these effectiveness measures could be computed across any gradient of coverage, |*f*_1_ − *f*_2_|, other than those with *f*_2_ = 0.

We base the indirect, overall, and total vaccine effectiveness on the reduction in infection rates when comparing the appropriate groups within a subregion with no vaccination to a comparable subregion with a fraction *f* > 0 of the population vaccinated (Figure 2). The direct effectiveness compares the vaccinated to the unvaccinated within a subregion (Figure 2). Since the vaccinated and unvaccinated in a particular subregion are subject to the same intensity of infection, the direct effectiveness should be independent of the vaccine coverage [15]. This condition held for the randomized vaccine trial; however, if in actual practice vaccinated people tend to have less risk of cholera infection than unvaccinated people due to factors other than vaccination itself, then the actual vaccine effectiveness measures would be attenuated. In addition, we assume that the vaccine induces immunity that results in a proportional reduction in the probability of infection per contact with an infectious source [15], i.e., a leaky vaccine. For our application, we further average over all the subregions within vaccination coverage strata.

Vaccines would need to contain antigens that are reactive or have cross-reactivity to current circulating strains of cholera in endemic areas [12]. These would include the Vibrio cholerae O1 El Tor biotype with both the Ogawa and Inaba serotypes, as well as the O139 serogroup that has been circulating since 1993.

Since we are modeling endemic cholera, we assume that there is significant partial population-level immunity due to past exposure to cholera. The actual level of such protection is complex and difficult to determine. Past analysis of cholera incidence in Matlab reveals that there is partial waning of population-level immunity over six months [12], although there is reason to believe that there is some partial long-term immunity as well [16,17]. Since we are modeling endemic cholera over a limited (one year) time horizon, we assume that infection in individuals results in complete immunity for the remainder of the simulation period, but this immunity would be partially lost with time. We also model populations that may not have as high a level of partial population natural immunity than that of Matlab. We do this by increasing the relative susceptibility of the simulation population in contrast to Matlab. For example, people in a population relatively new to cholera could have a susceptibility that is twice as high as that of Matlab per contact with an infectious source. In this case, the probability of infection per contact with an infectious source would be twice has high as that probability in Matlab. The relative susceptibility multiplier would be two in this case (see Text S3).

## Results

**Figure 3:**
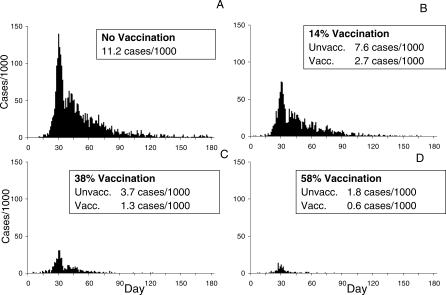
Simulated Number of Cholera Cases per 1,000 over a 180-Day Period in the Matlab Study Population for a Single Stochastic Realization (A) No vaccination. (B) 14% vaccination coverage of women and children. (C) 38% vaccination coverage. (D) 58% vaccination coverage.

We calibrated the simulation model using cholera incidence data observed in the first year of the vaccine trial (Table 1) over a 180 d period in order to capture all the cholera transmission during the large annual cholera outbreak. This was done by varying the transmission probability, π (see Figure S6 and Text S2), such that the differences between the observed incidence rates and the simulated incidence rates in Table 1 were minimized. The estimated reproductive number was 5.0 with a standard deviation of 3.3 (see Text S2). A summary of the parameters and their baseline values are shown in Table S1. The vaccine coverage levels in the target population and the effective coverage in the entire population from the trial are summarized in Table 1. We assume that vaccinated people receive an effective regimen of two doses. The observed cholera incidence rates among the unvaccinated ranged from a high of 7.0 cases/1,000 over 180 d for the subregions with the lowest coverage in the target population, centered at 14%, to 1.5 cases/1,000 for the highest coverage, centered at 58%. The observed cholera incidence rates among the vaccinated ranged from a high of 2.7 cases/1,000 for the subregions with the lowest coverage to 1.3 cases/1,000 for the highest coverage. We set the vaccine efficacy (VE) for susceptibility to VE*_S_* = 0.7 [2,3] and for infectiousness to VE*_I_* = 0.5. The simulated incidence rates provided a good fit to the data based on a χ^2^ goodness-of-fit test for frequency data (*p* = 0.84, 9 degrees of freedom). Figure 3A–3D show the number of cases over time comparing the unvaccinated to the vaccinated populations. Videos S1 and S2 show the spatial–temporal epidemics at different coverage levels. For effectiveness measures, we compare the intervention subregions to hypothetical subregions that receive no vaccine, i.e., *f* = 0. Table 2 shows the indirect, total, and overall effectiveness estimated by the model for possible coverage levels when comparing coverages in the entire population, 2 y of age and older, ranging from 10% to 90% compared to no vaccination. For example, the average indirect effectiveness, comparing a population with a coverage of 30% to one with no vaccination, is 70% (also see Figure S7). This indicates that on average, the cholera incidence among unvaccinated people in a population with 30% coverage would be reduced by 70% compared with a completely unvaccinated population. At this level of coverage, the total effectiveness of 90% indicates high protection for a vaccinated person in a population with 30% vaccination coverage, while the overall effectiveness of 76% indicates a good overall reduction in risk to the overall population. According to the model, around 40 cases of cholera are prevented per 1,000-dose regimens of vaccine at low coverage and 13 cases at high coverage. At coverage levels of 50% and higher, all levels of effectiveness exceed 85%, resulting in the nearly total control, i.e., an overall annual cholera incidence of about 1 case per 1,000 people, of cholera transmission.

**Table 1:**
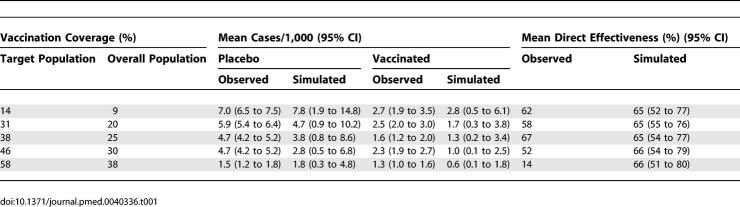
Vaccination Coverage, Average Incidence Rates, and Direct Effectiveness (Calibration Runs)

**Table 2:**
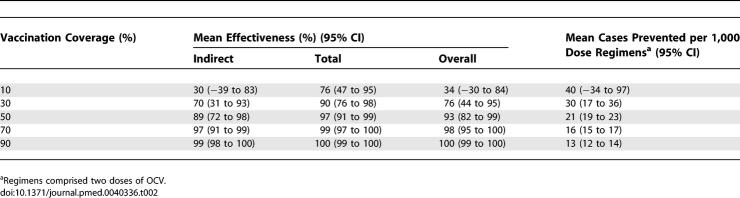
Average Indirect, Total, and Overall Effectiveness of Vaccination, and Cases Prevented Per 1,000-Dose Regimens

From Table 1, we see that the simulated direct effectiveness at all coverage levels is estimated from the simulations to be about 66%, while the vaccine efficacy for susceptibility, VE*_S_* is pre-set at 70%. This small underestimation is due to the fact that we model the vaccine effect to be a 70% reduction in the risk of infection per contact with an infective source, i.e., a leaky effect, but we use the risk ratio estimator of vaccine effectiveness over the entire cholera epidemic. We do this for the purpose of comparison, as this was the primary estimator used in the cholera vaccine trial in Matlab [2]. We have shown that an estimator based on the monthly hazard ratio gives a similar, but more accurate estimate of actual vaccine efficacy [3]. Also note from Table 1 that the observed estimate of the direct effectiveness is only 14% in the highest vaccination coverage category when it should be around 66%. This discrepancy probably reflects small sample bias due to the low cholera incidence in the highest vaccine coverage category.

**Figure 4:**
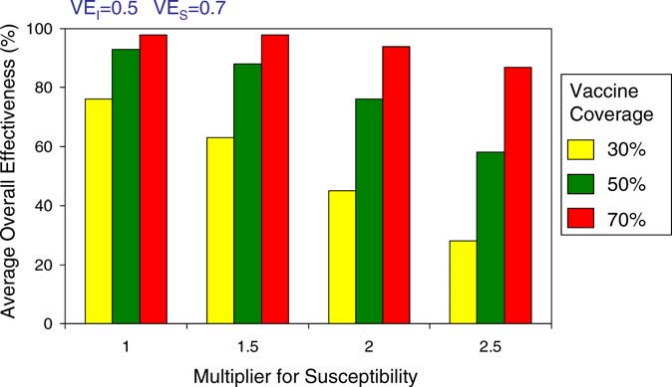
Average Overall Vaccine Effectiveness When Varying Relative Susceptibility These comparisons are for relative susceptibility in populations ranging from 1.5 to 2.5 times as susceptible as Matlab. The Matlab results are shown when the multiplier is 1. For populations that are 2–2.5 times as susceptible as Matlab, at least 70% vaccine coverage is needed to achieve an overall effectiveness of at least 80%. The vaccine efficacies are preset at VE*_S_* = 0.7 and VE*_I_* = 0.5.

Figure 4 shows the overall effectiveness estimated by the model for possible coverage levels in populations at different levels of relative susceptibility compared to Matlab. For populations that are 1.5 times as susceptible as those in Matlab, 50% coverage would still be sufficient to achieve an overall effectiveness of 80%. However, for populations that are 2–2.5 times as susceptible, 70% vaccine coverage would be necessary to achieve control.

Since vaccine efficacy can vary for different field settings and vaccines, a sensitivity analysis was carried out on the VE*_S_* and the VE*_I_* to determine the minimum efficacy needed to maintain control at the vaccine coverages explored above. The VE*_S_* and VE*_I_* were varied from 0.3 to 0.8 (Figures S8–S15). These analyses reveal that theVE*_S_* could be as low as 0.5 and the VE*_I_* as low as 0.3 to still maintain control of cholera, as long as the vaccine coverage were 50% or higher. Further sensitivity analysis on the vaccine coverage (Figures S13–S15) reveals that vaccine coverage should be at least 50% to maintain control.

Sensitivity analyses also were performed for values of the seasonal boost factor, the relative infectiousness of symptomatic infectives, and varied subregion sizes. The baseline epidemic with no vaccination was calibrated to the simulated cholera incidence data for Matlab with no vaccination. Our result that in populations like Matlab 50% vaccine coverage should be sufficient to control cholera remains valid for variation in the season boost factor (Figure S16) and relative infectiousness (Figure S17). However, we did see variation with respect to subregion size. For larger subregions (>6 km^2^ each), 50% vaccine coverage was sufficient for control. But for very small subregions (0.04 km^2^ each), the average overall vaccine effectiveness approaches 75% with a vaccination coverage of 70% (Figure S18). This lower effectiveness implies that random mass vaccination may not be effective in small subregions, and vaccination would have to concentrate on the small subregions where transmission is occurring. This result could be applicable to epidemic cholera in small, dense settings such as refugee camps where coverage would have to be high to control transmission.

## Discussion

The results of this modeling study indicate that 50% coverage with OCV could control cholera transmission in endemic areas such as Matlab, Bangladesh through a combination of direct and indirect effects.. At this level of coverage, the model predicts an 89% reduction in cholera cases even among the unvaccinated, and a 93% reduction overall in the entire population. These results would apply only where cholera is endemic and population levels of immunity are relatively high. According to our simulations, areas where susceptibility is 2–2.5 as great as Matlab would need to have vaccine coverage of at least 70% to achieve cholera control. Since vaccine-induced protection with current OCV begins to wane after about two years, populations would have to be vaccinated biennially. This could be done in advance of the cholera season for regions that have clear seasonality. Alternatively, environmental predictors of cholera outbreaks could be sampled in regions where such a prediction capacity exists, and then vaccination could take place in advance of expected outbreaks. In environmental studies of cholera outbreak predictors in rural Bangladesh, increases in cholera incidence can be predicted several weeks in advance by water temperature, water depth, rainfall, conductivity, and copepod counts [14]. Such predictors have been developed for Bangladesh and parts of Latin America, but also need to be developed for other regions with substantial risk, especially in Africa. Rapid mass vaccination could take place after such predictors indicate that outbreaks are likely. Further research needs to be carried out on environmental predictors of cholera incidence to make such an assessment reliable. For endemic cholera, population-level immunity is relatively high, making control possible with relatively low vaccine coverage levels. For epidemic cholera, where population level immunity may not be high, rapid vaccination could also be beneficial, but further study would be required to determine the higher coverage levels necessary to obtain substantial indirect protection.

The results of our analysis are based on a mathematical model closely calibrated to cholera incidence data from a large-scale cholera vaccine trial in Matlab, Bangladesh. We would hope that the model captures the dynamics of endemic cholera sufficiently to allow us to evaluate the effectiveness of cholera vaccination at different coverage levels in other settings. However, a major limitation of this research is that the results are only as good as the data the model is based on and the veracity of the modeling assumptions. Verification of the results needs to take place through further community cholera vaccine trials with different coverage levels in a variety of geographic locations. A number of such vaccine trials are currently being planned. The modeling methods presented here can be used to help guide the design, analysis, and interpretation of such trials. Based on the results of such trials, such a model can be used to aid in designing successful cholera vaccination strategies. A second limitation of the model is that we have not explicitly modeled the level of natural immunity in populations with endemic cholera, but rather we alter the per-contact transmission probability to reflect different levels of immunity. This means that we can only approximate the effects of such immunity. More study is needed in this area.

We have not explicitly addressed how to target vaccines during a mass cholera vaccination campaign. In endemic cholera regions, partial immunity tends to increase with increasing age, as partially reflected in higher cholera incidence rates among the young [6,10]. In addition, it could take up to a month to vaccinate large populations. Although we would recommend vaccinating all age groups 2 y and over, with limited quantities of vaccine or time for administration, we would recommend the targeted vaccination of children, especially the very young.

The present analysis implies that mass immunization with killed, whole-cell based oral cholera vaccines could possibly confer a major protective impact against cholera in an endemic setting, even with modest levels of vaccine coverage, due to the combination of direct and indirect vaccine protective effects. It might, however, be questioned whether mass immunization with such vaccines is logistically feasible and affordable. Feasibility of delivery of two-dose regimens of B subunit–killed whole cell or whole cell-only oral cholera vaccines has been demonstrated in a stable refugee setting and a densely populated urban area in sub-Saharan Africa [18,19], as well as in an urban setting in Asia [4]. Although the cost of the B subunit–killed whole cell vaccine, which is produced by an international producer, may currently impede its use in developing country settings, we believe that a low-cost vaccine could be made available in the near future, pending regulatory issues. For example, the cost of purchase and delivery of a two-dose regimen of a currently available oral killed whole cell-only vaccine produced in Vietnam was only US$0.89 in Vietnam [4]. There is currently only one oral cholera vaccine, Dukoral, a B subunit–killed whole cell vaccine, in production and internationally licensed for people 2 y of age and older. This vaccine could be used in mass vaccination campaigns such as that undertaken against epidemic cholera in Mozambique in 2003–2004, with or without targeting of young children. The success of the vaccination campaign in Mozambique against a cholera epidemic in an endemic region demonstrates that cholera could be controlled with a relatively modest financial investment and an organized global surveillance and vaccination program. However, the cholera situation in particular regions should be carefully analyzed and modeled to investigate the coverages and vaccination strategies that would work best.

## Supporting Information

Figure S1 Overview of the Stochastic Simulator (19 KB PPT)

Figure S2 Age Distribution of the Input Population (168 KB PPT)

Figure S3 Bari Size Distribution (35 KB PPT)

Figure S4 Maps and Schematic of Bangladesh and the Matlab Study Area (A) Map of Bangladesh, showing location of Matlab. Matlab is in the Chandpur district of Bangladesh. It is located about 55 kilometers southeast of the country's capital, Dhaka at 23.38° north latitude and 90.72° east longitude.
(B) Close-up of Chandpur district, within which Matlab is contained.
(C) Rectangular grid mapped onto the Matlab region. The total area of the grid was approximately 384 km^2^. This area was divided into 64 rectangular subregions of approximately 6 km^2^ each. Study baris were contained within 43 of these subregions, shown in yellow in the figure.
(182 KB PPT)

Figure S5 Map Showing the Spatial Distribution of Baris Modeled in the Study (40 KB PPT)

Figure S6 Epidemic Curves and Average Incidence Rates for Calibration Runs Calibration runs were performed with 0% (A), 30% (B), 50% (C), and 70% (D) vaccination coverage in the entire population 2 y and older in age.
(82 KB PPT)

Figure S7 Average Cholera Incidence Rates for Different Vaccine Coverage Levels These simulations are for scenarios with vaccine coverage in the entire population (2 y and older in age) ranging from 30% to 70%. The solid line shows the average incidence among unvaccinated people, the dashed line among vaccinated people.
(43 KB PPT)

Figure S8 Effect on Incidence When Varying the VE_S_ and Vaccine Coverage The solid lines show the average incidence among unvaccinated people, the dashed lines among vaccinated people.
(53 KB PPT)

Figure S9 Effect on Average Indirect Vaccine Effectiveness When Varying the VE*_S_* and Vaccine Coverage (41 KB PPT)

Figure S10 Effect on Average Total Vaccine Effectiveness When Varying the VE*_S_* and Vaccine Coverage (37 KB PPT)

Figure S11 Effect on Average Overall Vaccine Effectiveness When Varying the VE*_S_* and Vaccine Coverage (41 KB PPT)

Figure S12 Effect on Incidence When Varying the VE*_I_* and Vaccine Coverage The solid lines show the average incidence among unvaccinated people, the dashed lines among vaccinated people.
(48 KB PPT)

Figure S13 Effect on Average Indirect Vaccine Effectiveness When Varying the VE*_I_* and Vaccine Coverage (41 KB PPT)

Figure S14 Effect on Average Total Vaccine Effectiveness When Varying the VE*_I_* and Vaccine Coverage (41 KB PPT)

Figure S15 Effect on Average Overall Vaccine Effectiveness When Varying the VE*_I_* and Vaccine Coverage (42 KB PPT)

Figure S16 Effect on Average Overall Vaccine Effectiveness When Varying the Seasonal Boost (43 KB PPT)

Figure S17 Effect on Average Overall Vaccine Effectiveness When Varying the Relative Infectiousness of Symptomatic Infectives (43 KB PPT)

Figure S18 Effect on Average Overall Vaccine Effectiveness When Varying Subregion Sizes (42 KB PPT)

Table S1 Model Parameters and Functions (33 KB DOC)

Text S1 Simulator Overview and Population Structure (34 KB DOC)

Text S2 Infection Function (45 KB DOC)

Text S3 Model Calibration and Sensitivity Analyses (34 KB DOC)

Video S1 Animated Maps of Matlab Show the Spatial–Temporal Distribution of Cholera Cases over the First 100 Days of Cholera Epidemics when 0%, 10%, and 30% Vaccination Coverage Is Implemented Each animation is based on a single, representative run of the simulator. For each coverage level, the map shows red dots, indicating baris with at least one cholera case, and yellow dots, indicating baris in which cases occurred but have recovered or died. Beneath each animated map is the corresponding animated epidemic curve, showing the number of cholera cases occurring each day.
(6.3 MB AVI)

Video S2 Animated Maps of Matlab Show the Spatial–Temporal Distribution of Cholera Cases over the First 100 Days of Cholera Epidemics when 50%, 70%, and 90% Vaccination Coverage Is Implemented Each animation is based on a single, representative run of the simulator. For each coverage level, the map shows red dots, indicating baris with at least one cholera case, and yellow dots, indicating baris in which cases occurred but have recovered or died. Beneath each animated map is the corresponding animated epidemic curve, showing the number of cholera cases occurring each day.
(4.0 MB AVI)

## Abbreviations

CI: confidence interval
ICDDR,B: International Centre for Diarrhoeal Disease Research, Bangladesh
Research: Bangladesh OCV, oral cholera vaccine
VE: vaccine efficacy
