# Supplementary material for: Controlling Endemic Cholera with Oral Vaccines
Source: PLoS Med. 2007 Nov 27;4(11):e336. doi: 10.1371/journal.pmed.0040336 (PMC2082648; doi:10.1371/journal.pmed.0040336)
Supplement: Figure S1 — (19 KB PPT) [file pmed.0040336.sg001.ppt]

## Slide 1
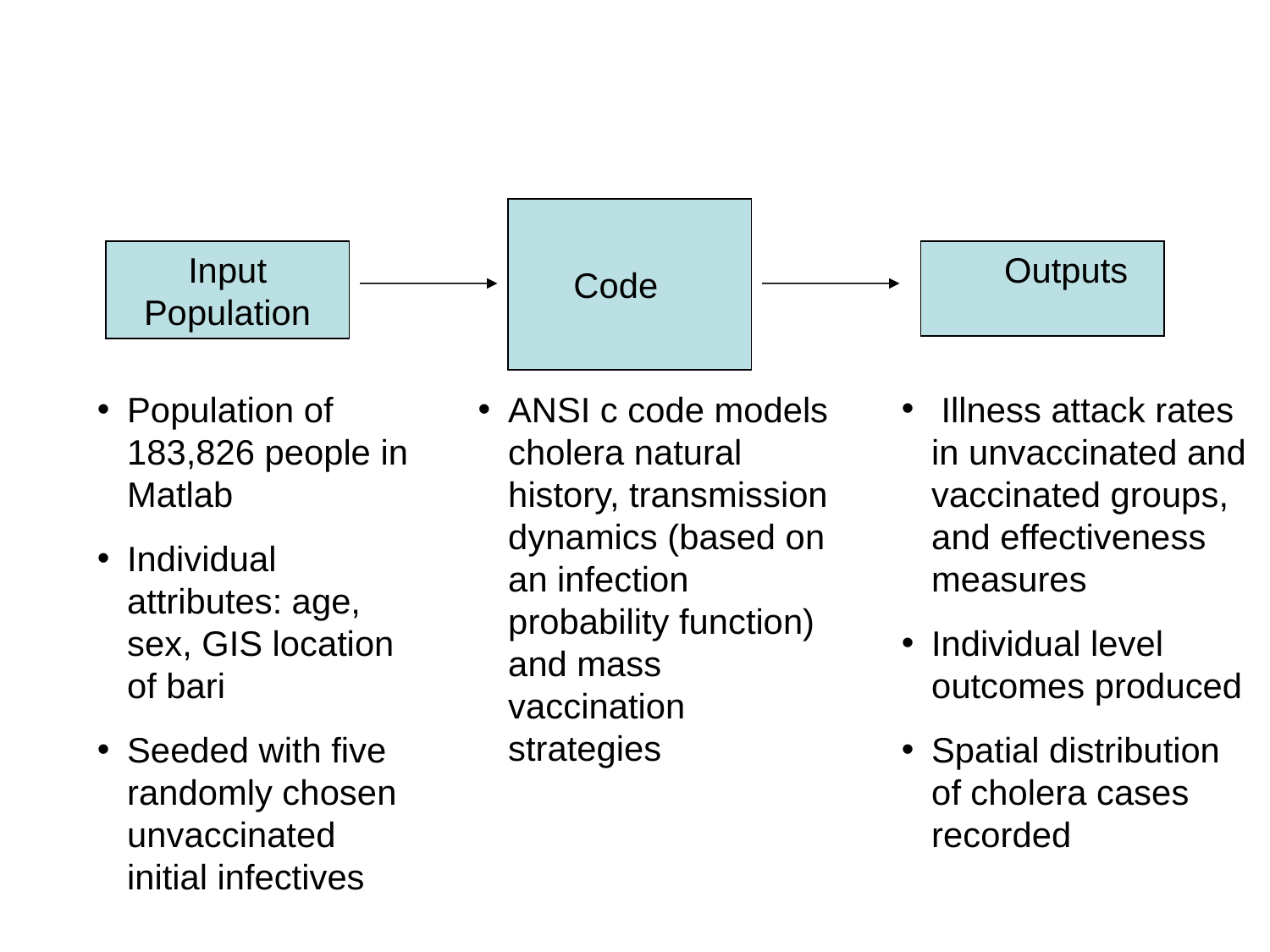

Code
# Outputs
Input Population
Population of 183,826 people in Matlab
Individual attributes: age, sex, GIS location of bari
Seeded with five randomly chosen unvaccinated initial infectives
ANSI c code models cholera natural history, transmission dynamics (based on an infection probability function) and mass vaccination strategies
 Illness attack rates in unvaccinated and vaccinated groups, and effectiveness measures
Individual level outcomes produced
Spatial distribution of cholera cases recorded
