# Supplementary material for: Controlling Endemic Cholera with Oral Vaccines
Source: PLoS Med. 2007 Nov 27;4(11):e336. doi: 10.1371/journal.pmed.0040336 (PMC2082648; doi:10.1371/journal.pmed.0040336)
Supplement: Figure S2 — (168 KB PPT) [file pmed.0040336.sg002.ppt]

## Slide 1
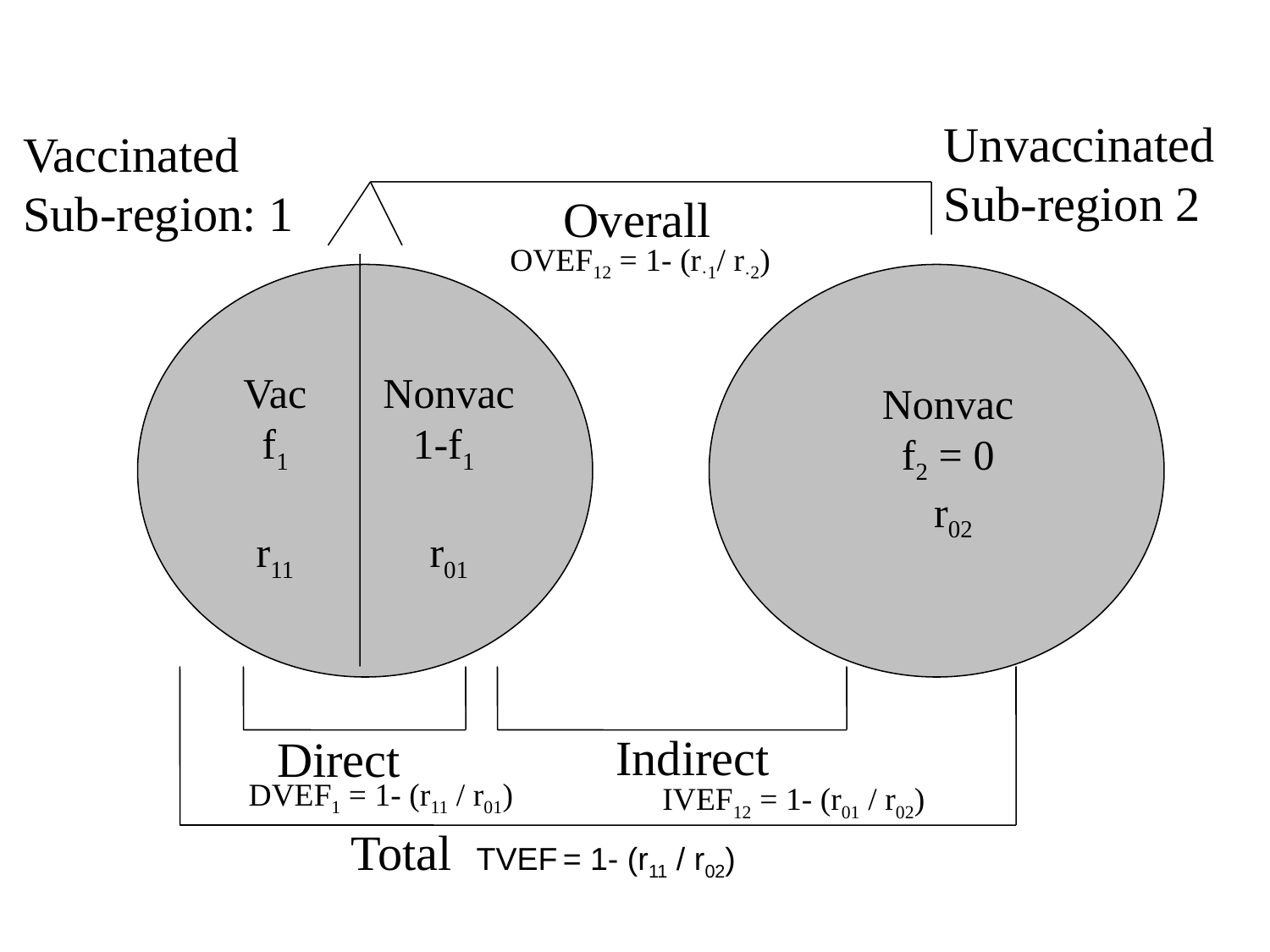

Unvaccinated
Sub-region 2
Vaccinated
Sub-region: 1
Overall
OVEF12 = 1- (r·1/ r·2)
Vac
f1
r11
Nonvac
1-f1
r01
Nonvac
f2 = 0
 r02
Indirect
Direct
DVEF1 = 1- (r11 / r01)
IVEF12 = 1- (r01 / r02)
Total TVEF = 1- (r11 / r02)
