# Supplementary figures and images for: Controlling Endemic Cholera with Oral Vaccines
Source: PLoS Med. 2007 Nov 27;4(11):e336. doi: 10.1371/journal.pmed.0040336 (PMC2082648; doi:10.1371/journal.pmed.0040336)

## Slide 1
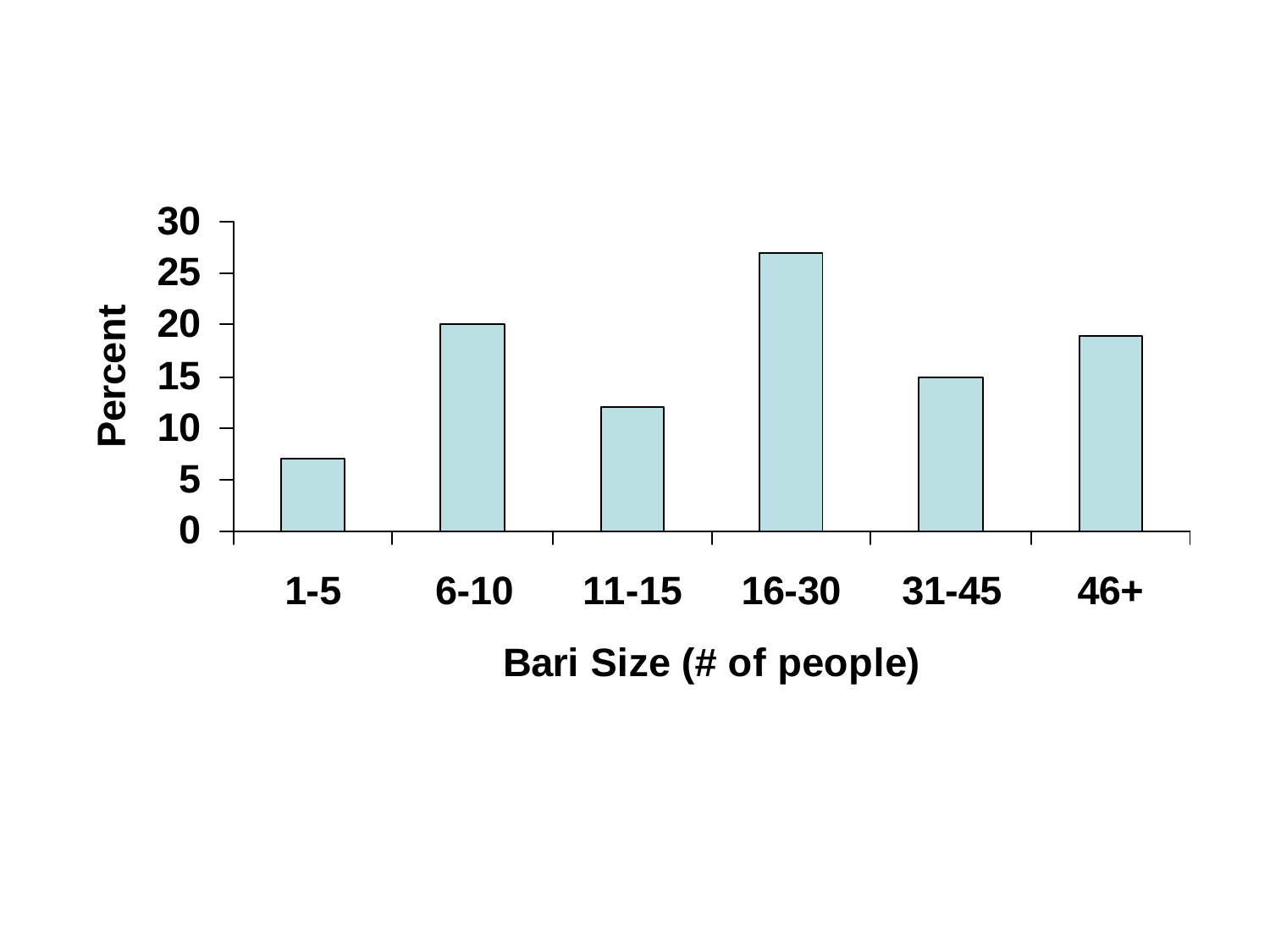

Supplement: Figure S3 — (35 KB PPT) [file pmed.0040336.sg003.ppt]

## Slide 1
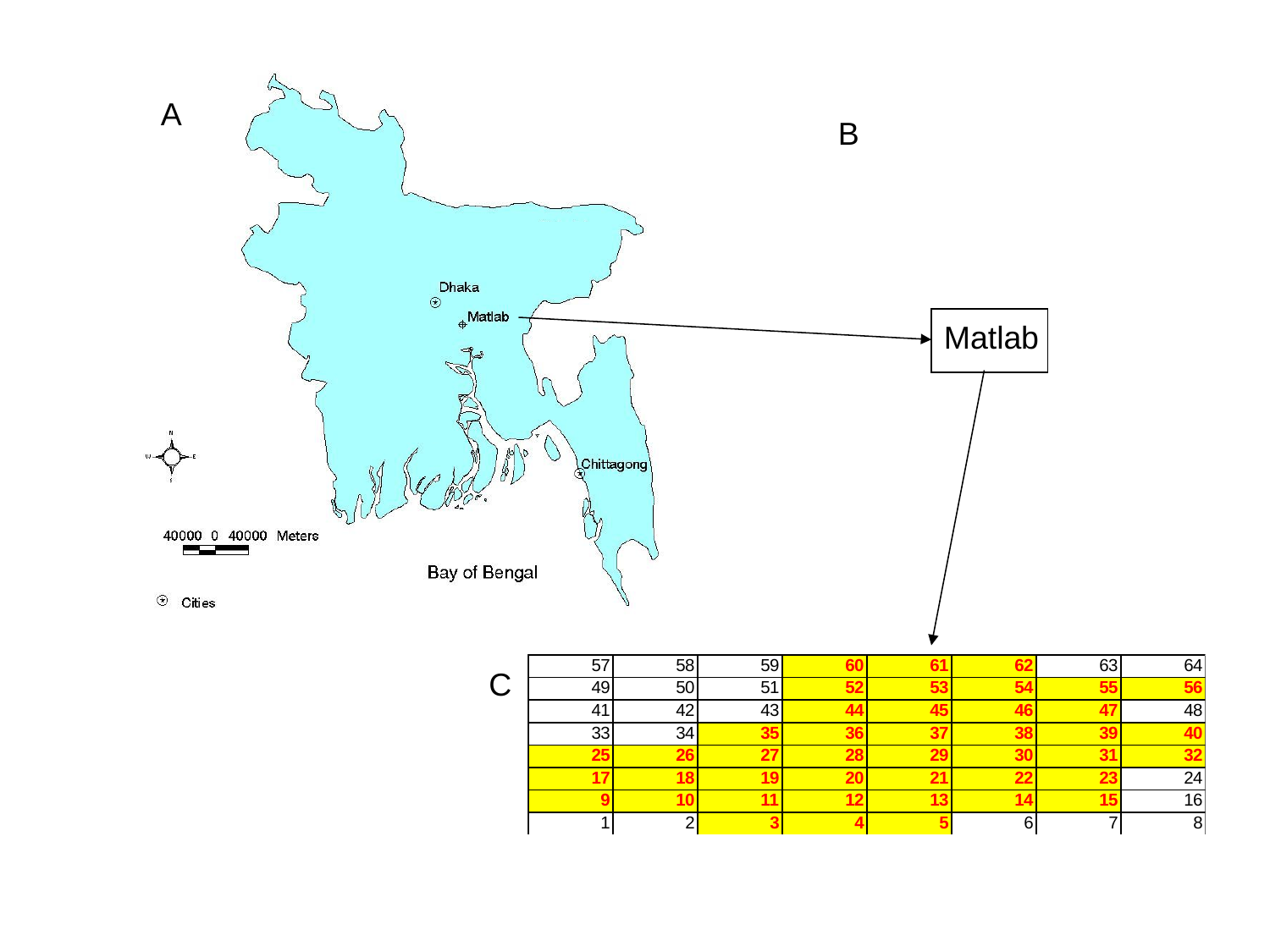

A
B
Matlab
C

Supplement: Figure S4 — (A) Map of Bangladesh, showing location of Matlab. Matlab is in the Chandpur district of Bangladesh. It is located about 55 kilometers southeast of the country's capital, Dhaka at 23.38° north latitude and 90.72° east longitude. (B) Close-up of Chandpur district, within which Matlab is contained. (C) Rectangular grid mapped onto the Matlab region. The total area of the grid was approximately 384 km2. This area was divided into 64 rectangular subregions of approximately 6 km2 each. Study baris were contained within 43 of these subregions, shown in yellow in the figure. (182 KB PPT) [file pmed.0040336.sg004.ppt]

## Slide 1
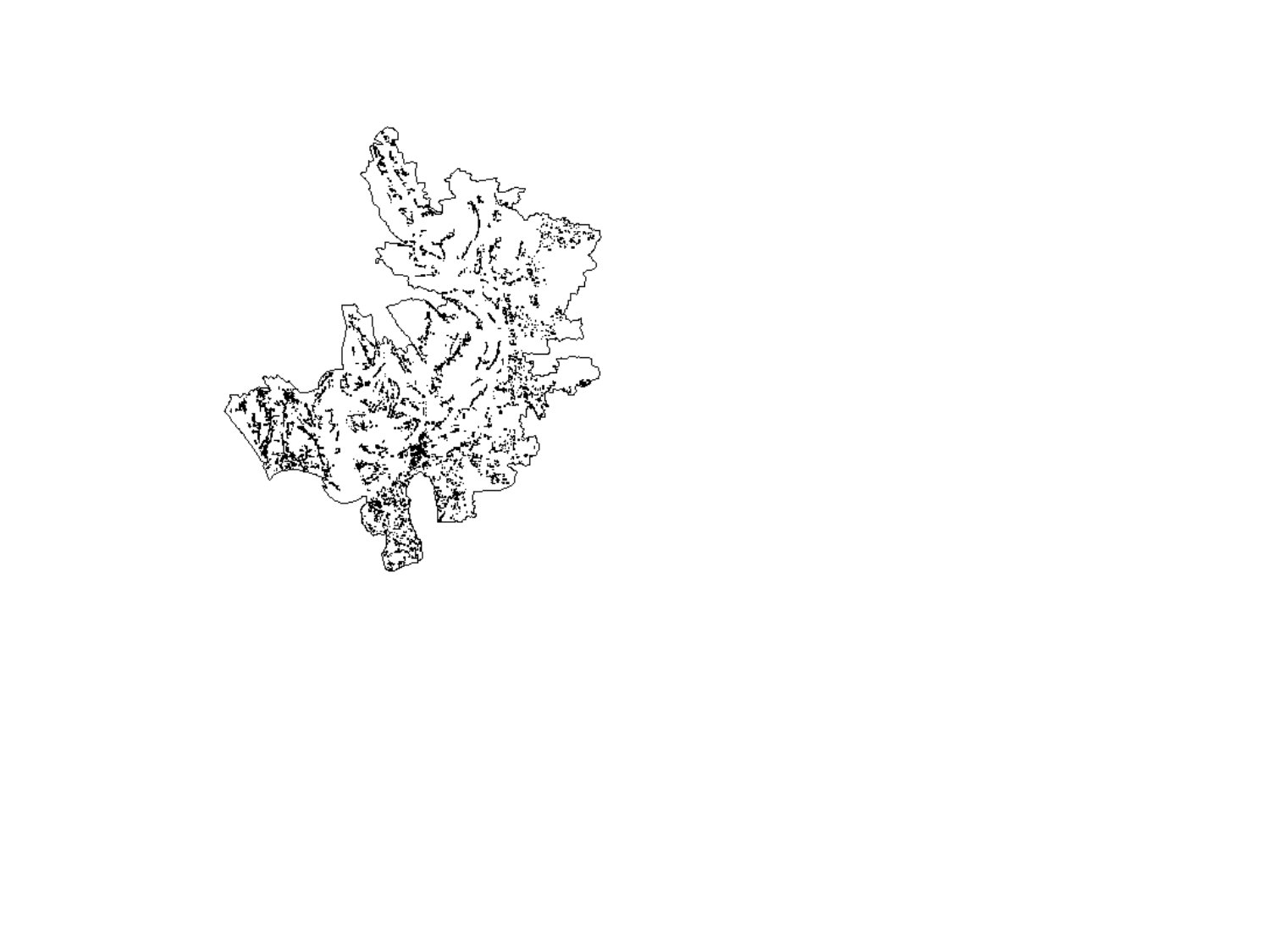

Supplement: Figure S5 — (40 KB PPT) [file pmed.0040336.sg005.ppt]

## Slide 1
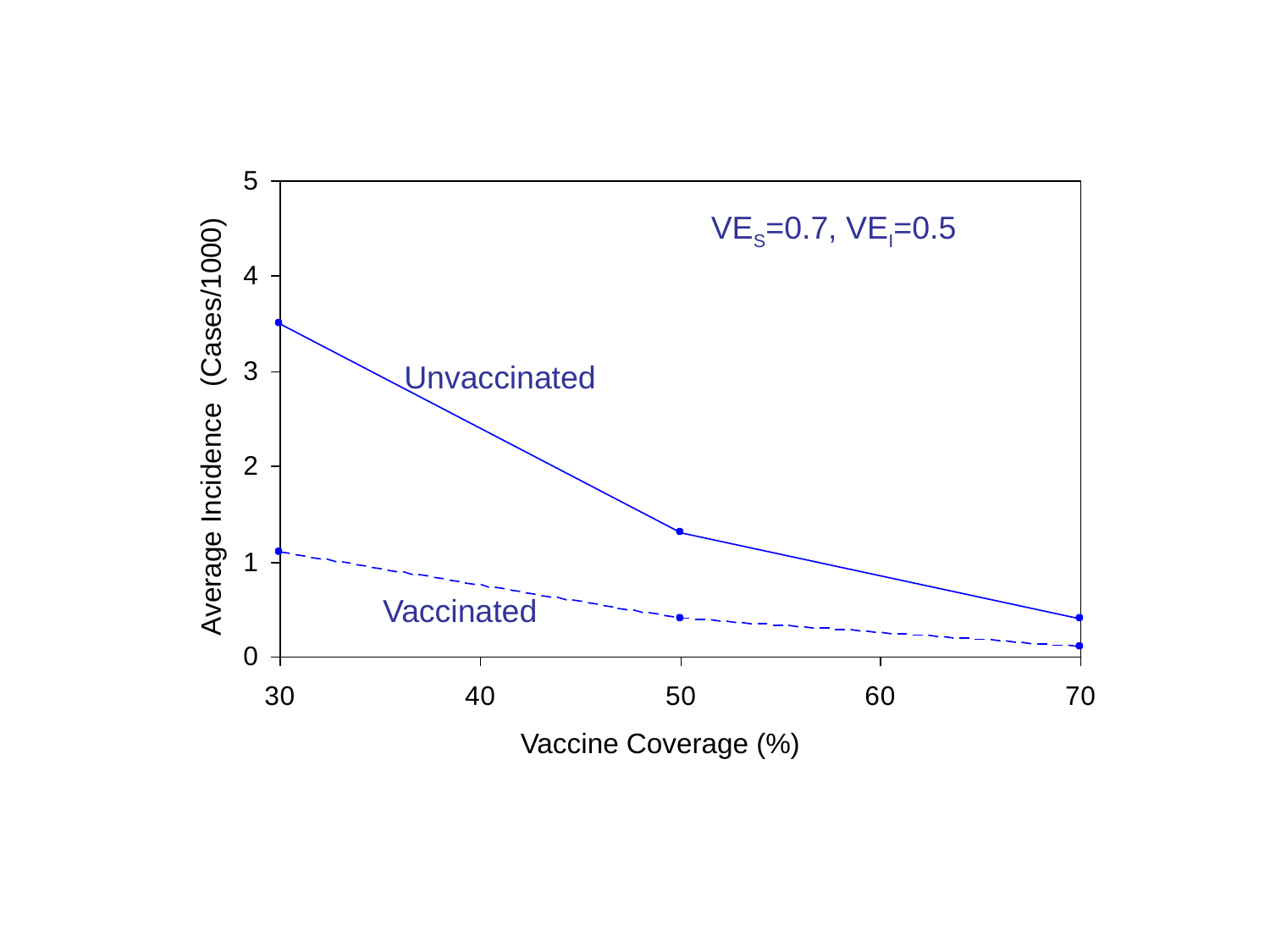

VES=0.7, VEI=0.5
Unvaccinated
 Average Incidence (Cases/1000)
Vaccinated
Vaccine Coverage (%)

Supplement: Figure S7 — These simulations are for scenarios with vaccine coverage in the entire population (2 y and older in age) ranging from 30% to 70%. The solid line shows the average incidence among unvaccinated people, the dashed line among vaccinated people. (43 KB PPT) [file pmed.0040336.sg007.ppt]

## Slide 1
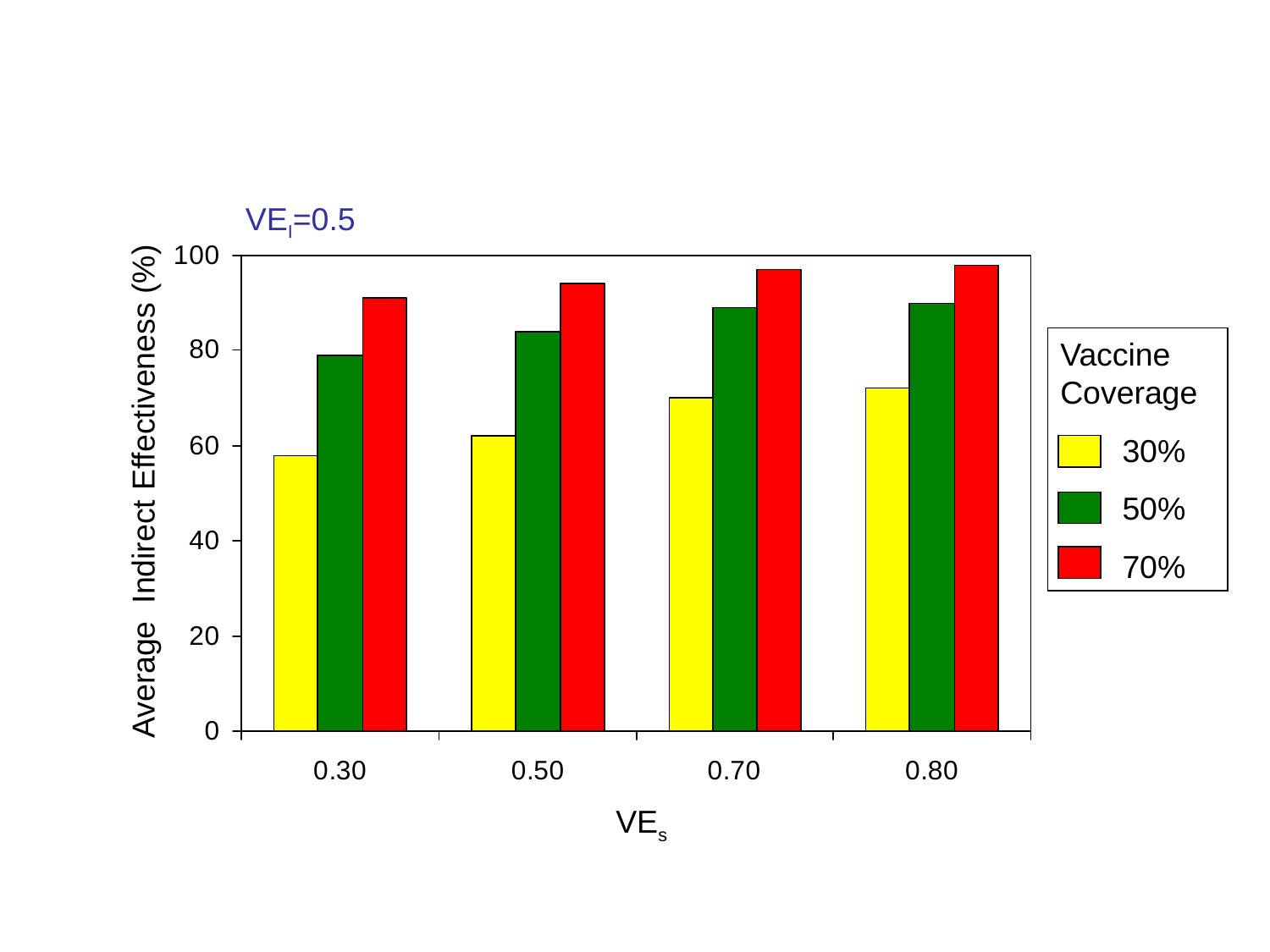

VEI=0.5
Vaccine Coverage
 30%
 50%
 70%
Average Indirect Effectiveness (%)
VEs

Supplement: Figure S9 — (41 KB PPT) [file pmed.0040336.sg009.ppt]

## Slide 1
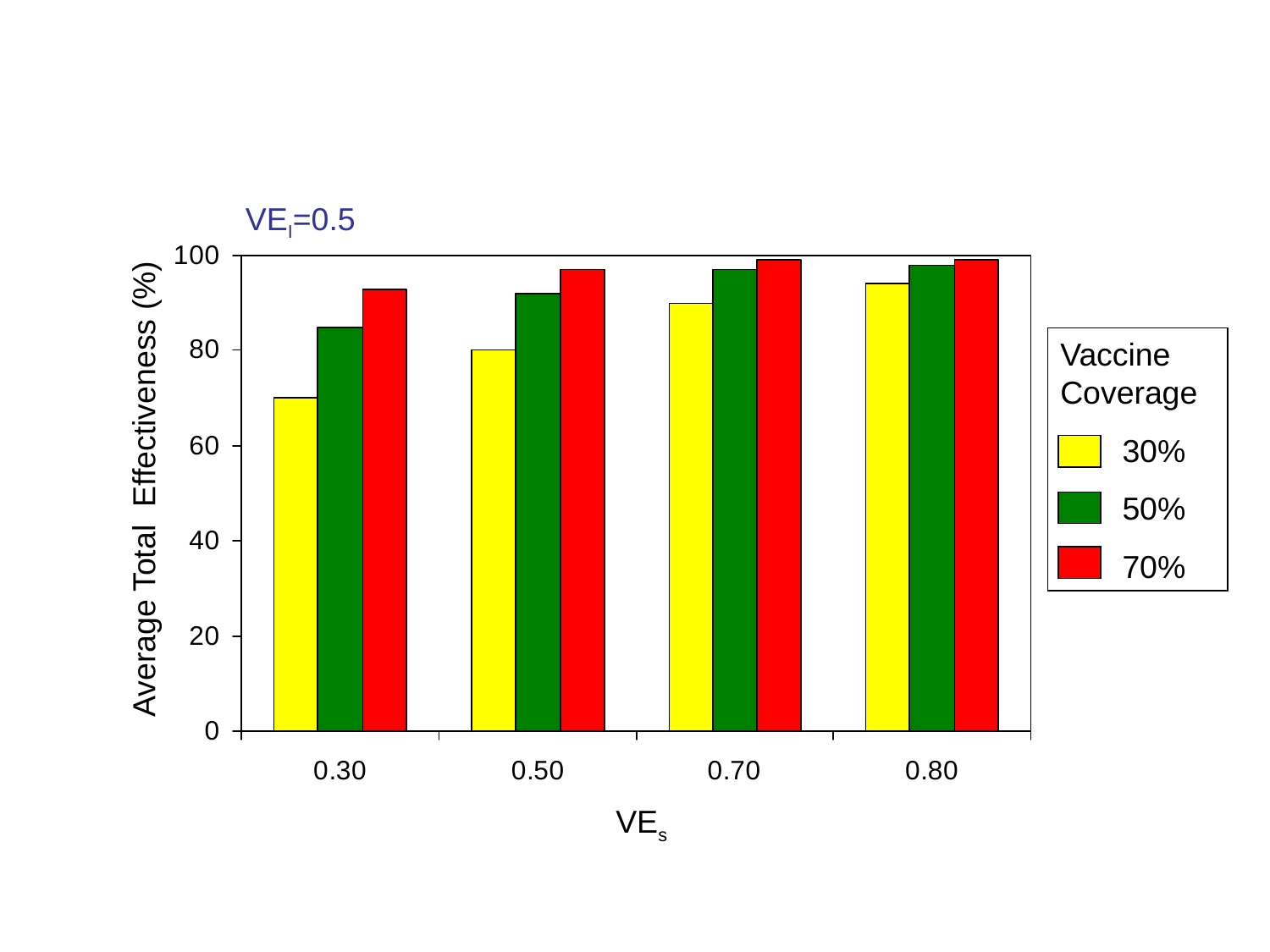

VEI=0.5
Vaccine Coverage
 30%
 50%
 70%
Average Total Effectiveness (%)
VEs

Supplement: Figure S10 — (37 KB PPT) [file pmed.0040336.sg010.ppt]

## Slide 1
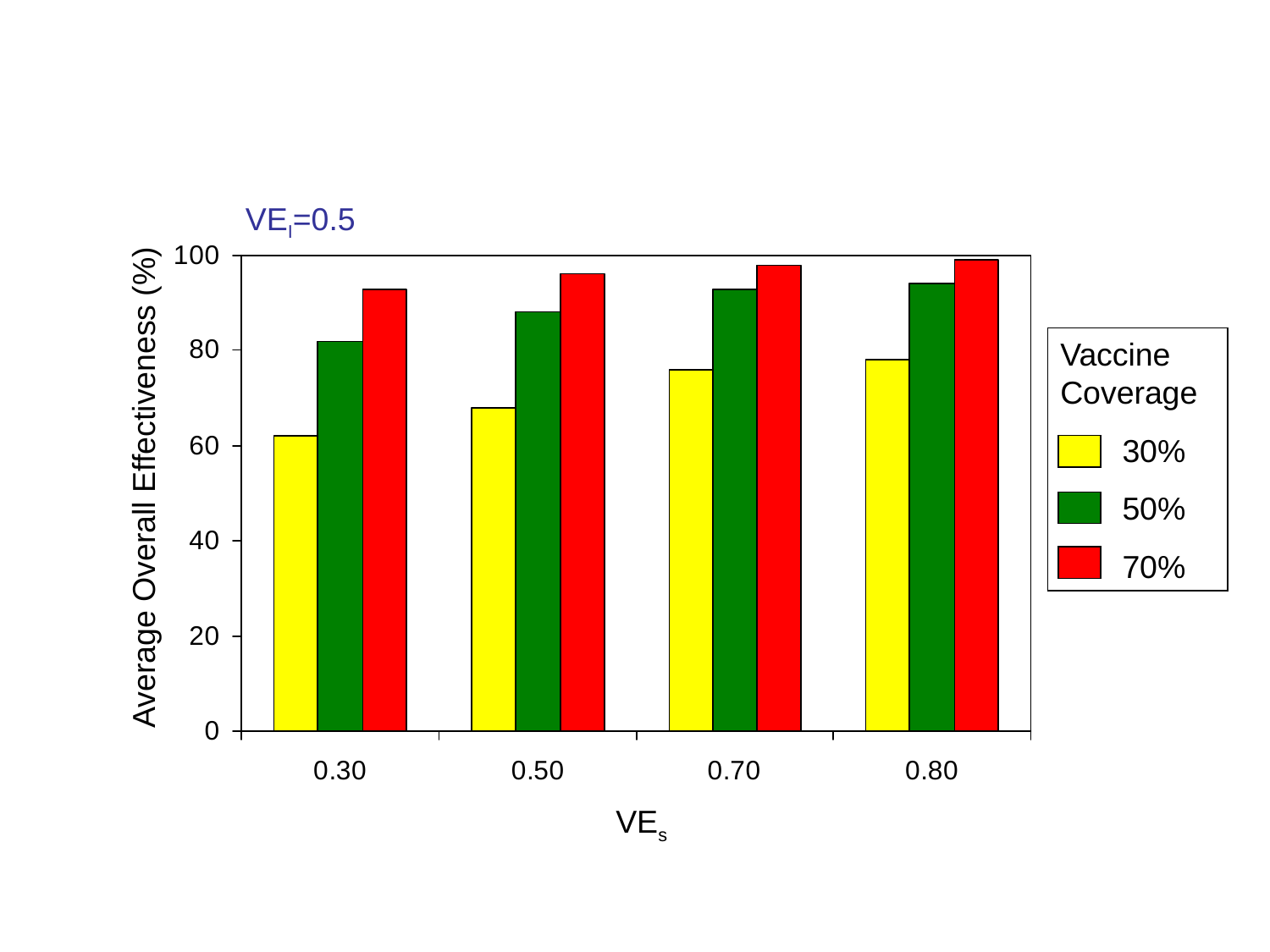

VEI=0.5
Vaccine Coverage
 30%
 50%
 70%
Average Overall Effectiveness (%)
VEs

Supplement: Figure S11 — (41 KB PPT) [file pmed.0040336.sg011.ppt]

## Slide 1
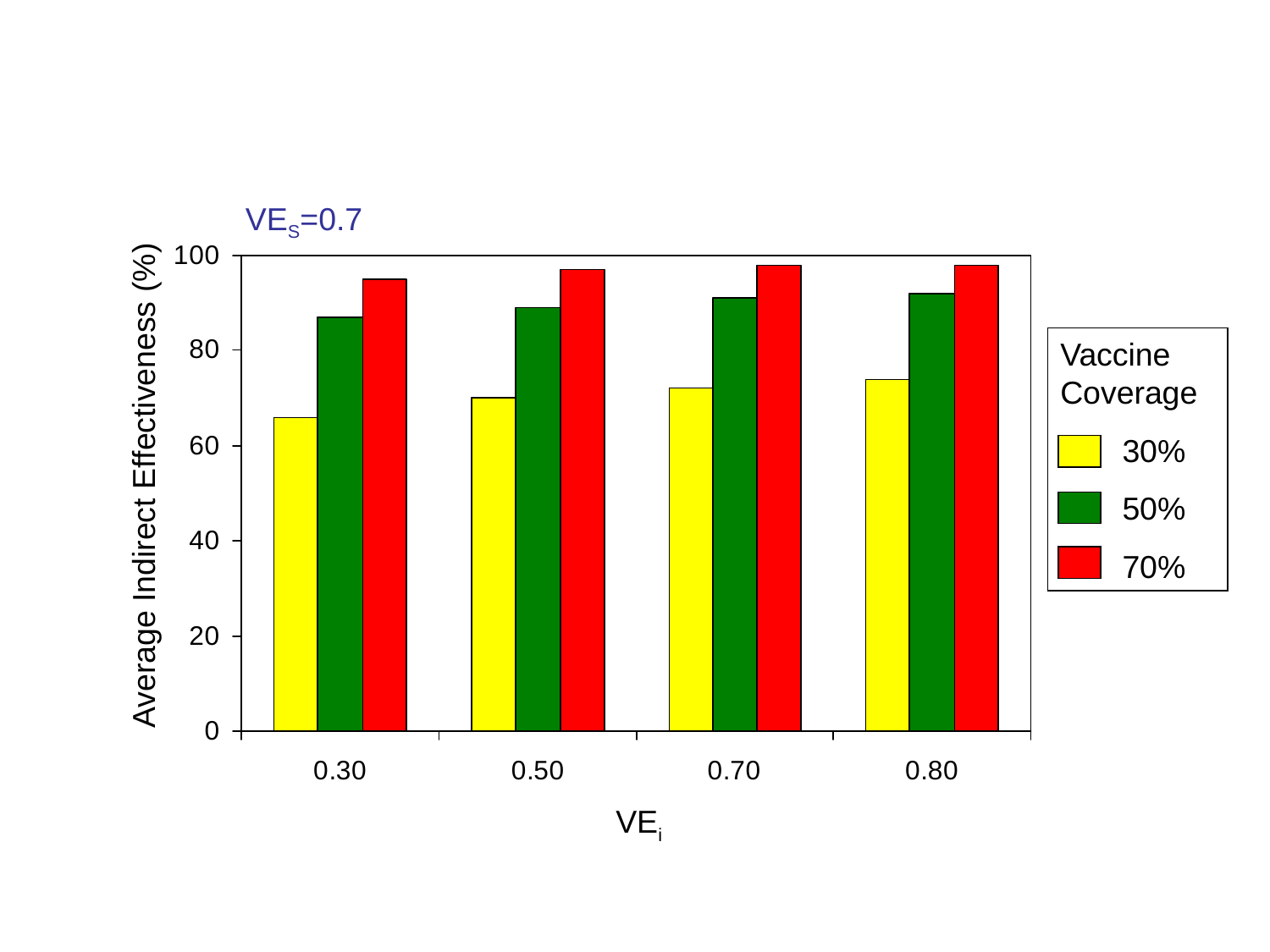

VES=0.7
Vaccine Coverage
 30%
 50%
 70%
Average Indirect Effectiveness (%)
VEi

Supplement: Figure S13 — (41 KB PPT) [file pmed.0040336.sg013.ppt]

## Slide 1
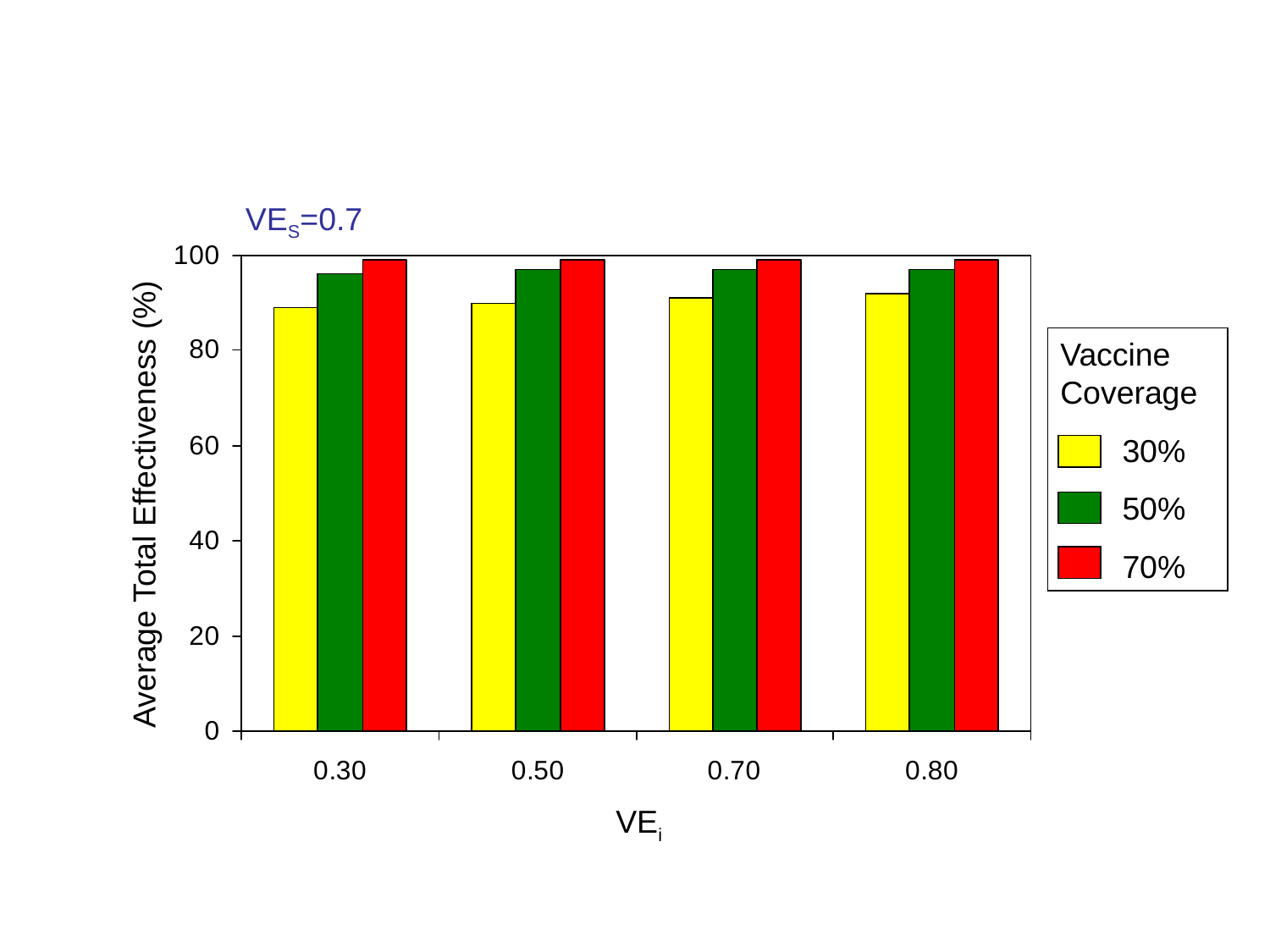

VES=0.7
Vaccine Coverage
 30%
 50%
 70%
Average Total Effectiveness (%)
VEi

Supplement: Figure S14 — (41 KB PPT) [file pmed.0040336.sg014.ppt]

## Slide 1
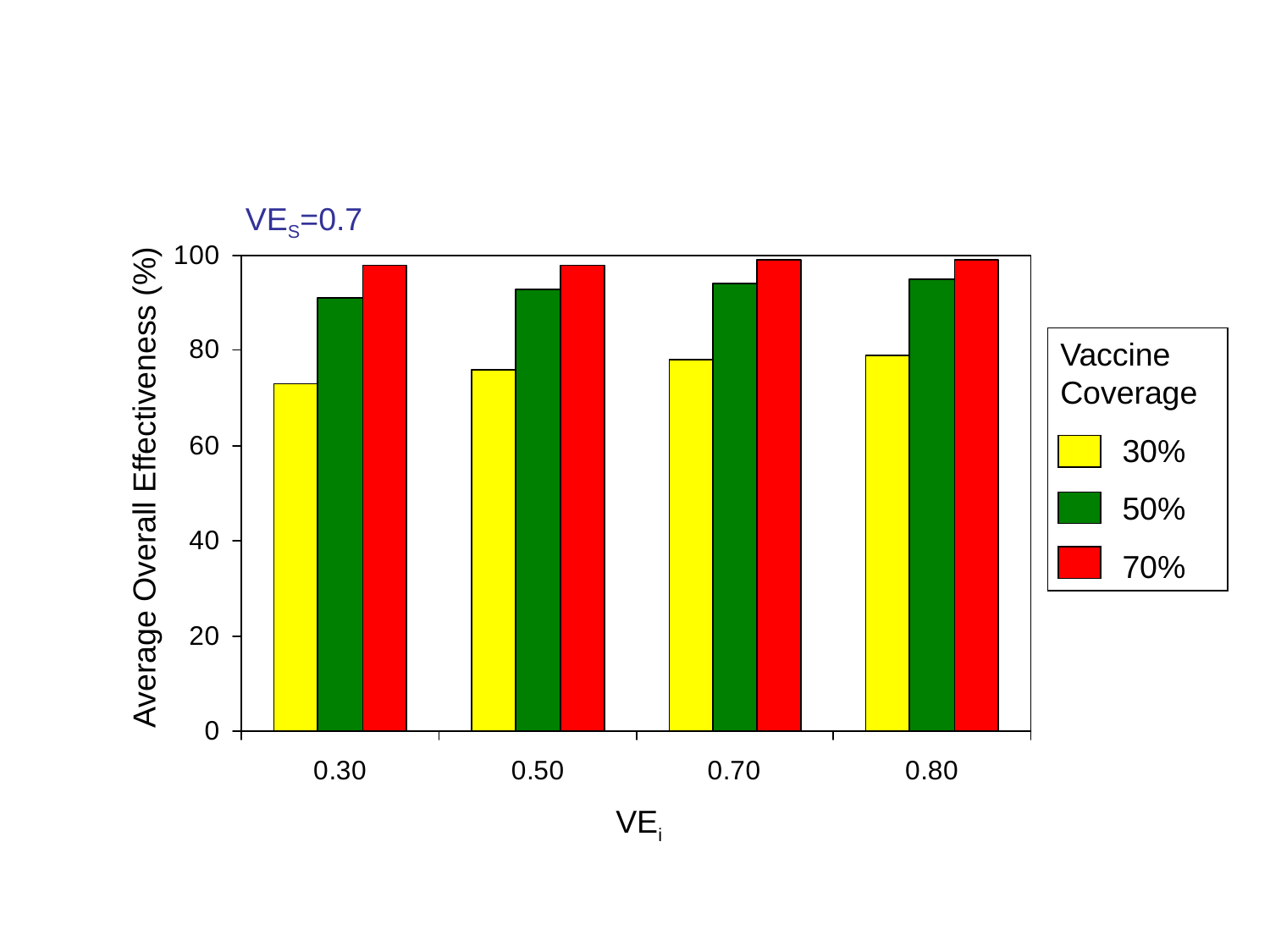

VES=0.7
Vaccine Coverage
 30%
 50%
 70%
Average Overall Effectiveness (%)
VEi

Supplement: Figure S15 — (42 KB PPT) [file pmed.0040336.sg015.ppt]

## Slide 1
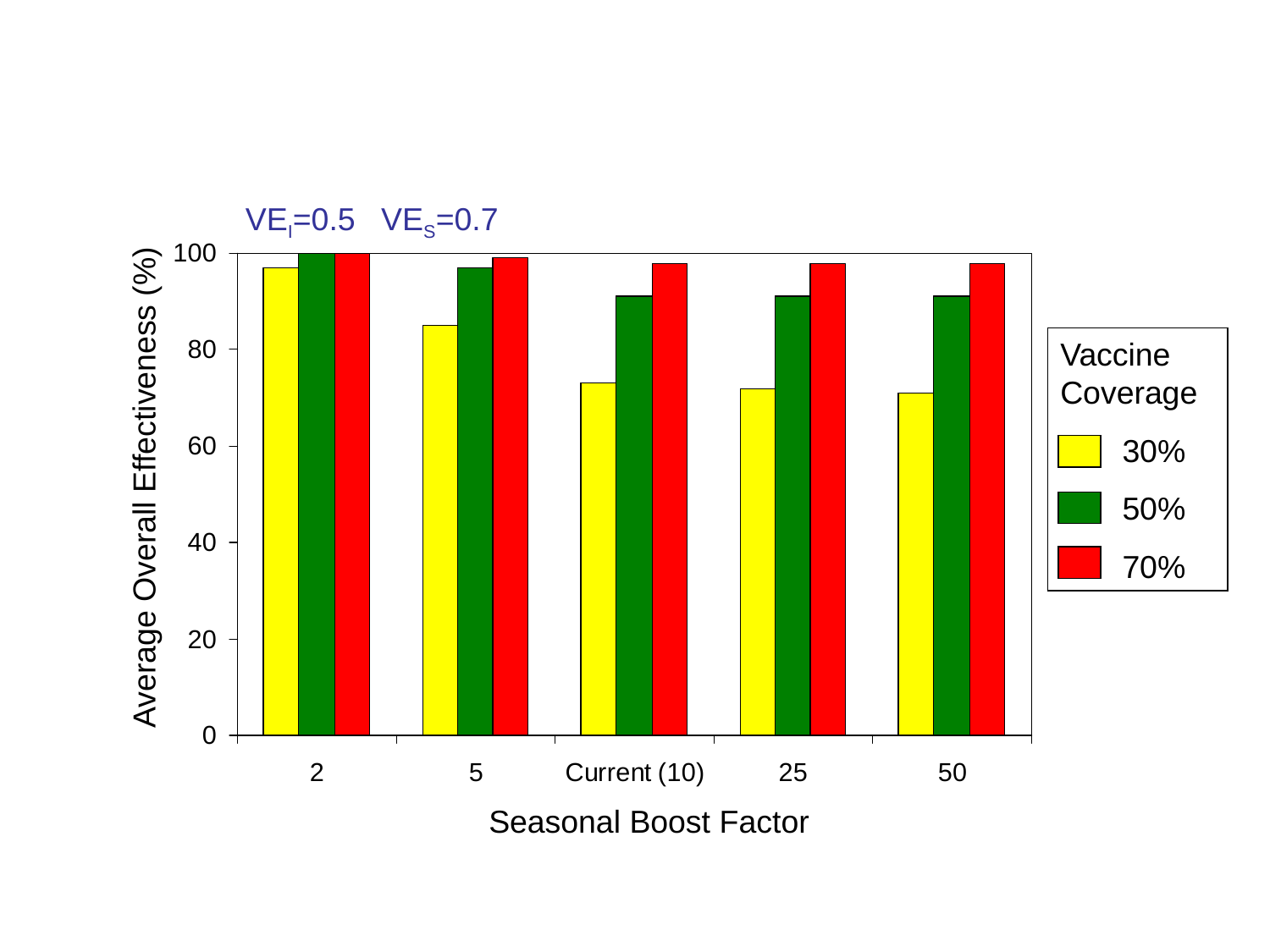

VEI=0.5	 VES=0.7
Vaccine Coverage
 30%
 50%
 70%
Average Overall Effectiveness (%)
Seasonal Boost Factor

Supplement: Figure S16 — (43 KB PPT) [file pmed.0040336.sg016.ppt]

## Slide 1
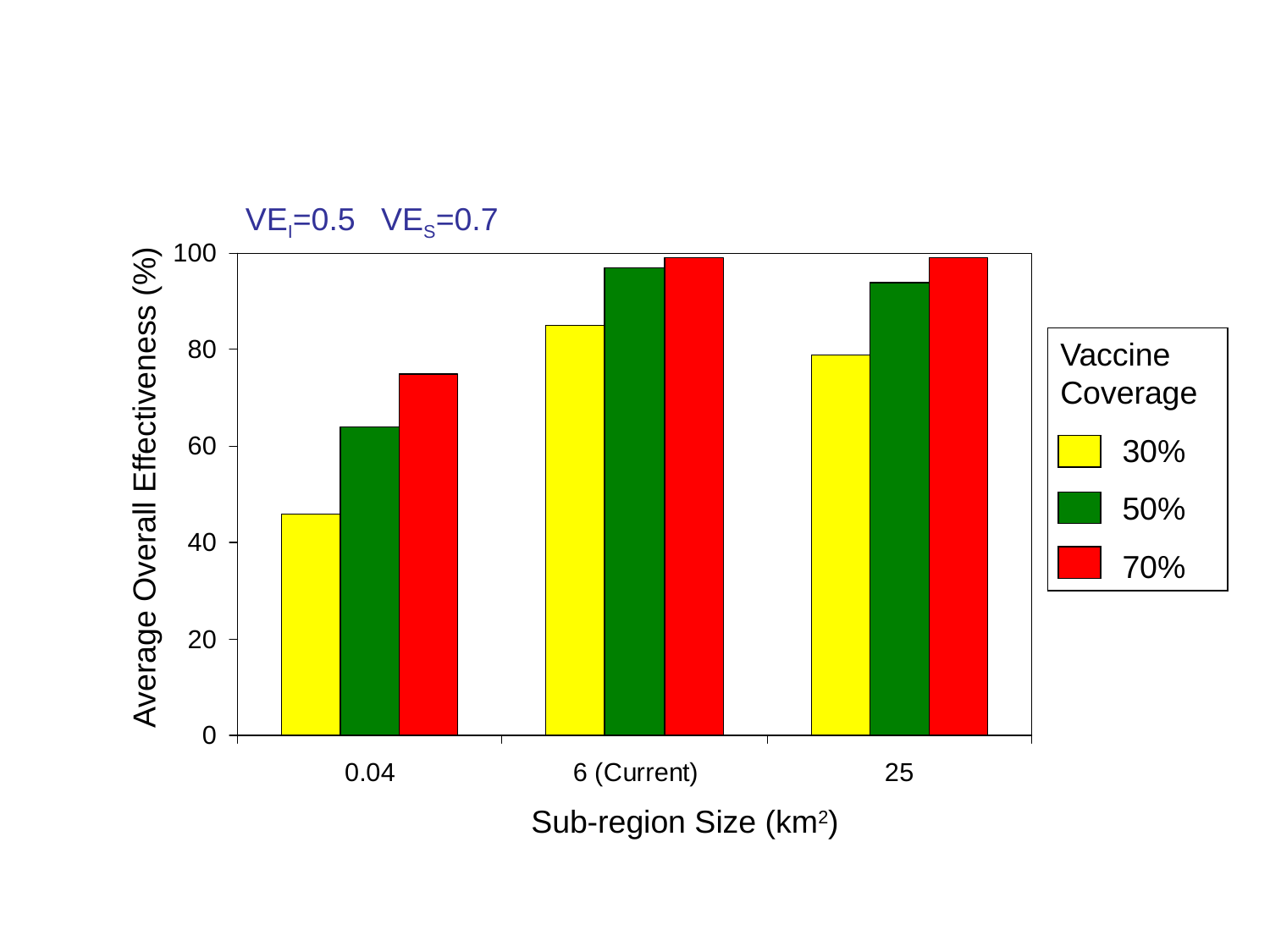

VEI=0.5	 VES=0.7
Vaccine Coverage
 30%
 50%
 70%
Average Overall Effectiveness (%)
Sub-region Size (km2)

Supplement: Figure S18 — (42 KB PPT) [file pmed.0040336.sg018.ppt]
