# Supplementary material for: Controlling Endemic Cholera with Oral Vaccines
Source: PLoS Med. 2007 Nov 27;4(11):e336. doi: 10.1371/journal.pmed.0040336 (PMC2082648; doi:10.1371/journal.pmed.0040336)
Supplement: Figure S6 — Calibration runs were performed with 0% (A), 30% (B), 50% (C), and 70% (D) vaccination coverage in the entire population 2 y and older in age. (82 KB PPT) [file pmed.0040336.sg006.ppt]

## Slide 1
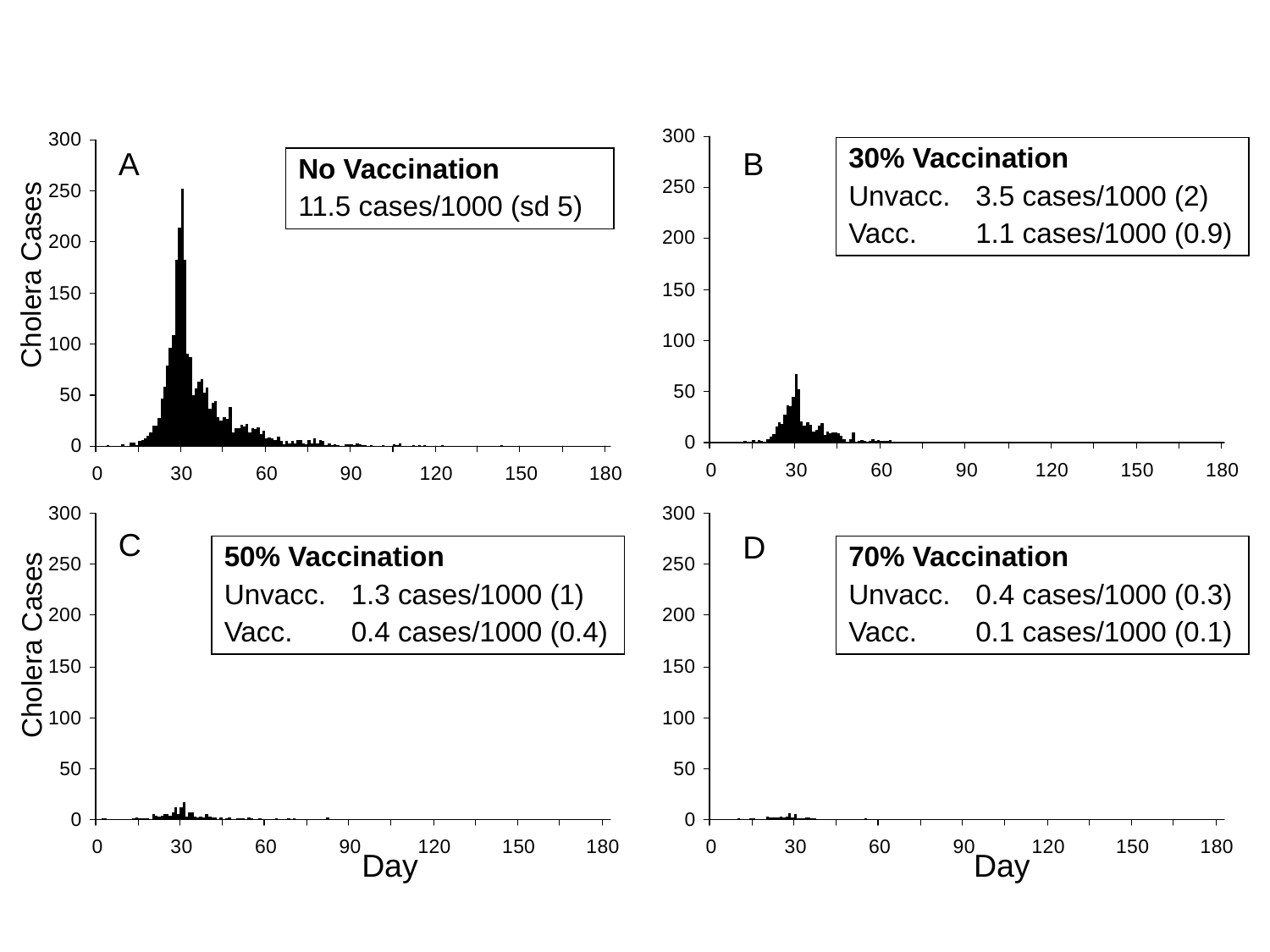

A
B
30% Vaccination
Unvacc.	3.5 cases/1000 (2)
Vacc.	1.1 cases/1000 (0.9)
No Vaccination
11.5 cases/1000 (sd 5)
Cholera Cases
C
D
50% Vaccination
Unvacc.	1.3 cases/1000 (1)
Vacc.	0.4 cases/1000 (0.4)
70% Vaccination
Unvacc.	0.4 cases/1000 (0.3)
Vacc.	0.1 cases/1000 (0.1)
Cholera Cases
Day
Day
