# Supplementary material for: Controlling Endemic Cholera with Oral Vaccines
Source: PLoS Med. 2007 Nov 27;4(11):e336. doi: 10.1371/journal.pmed.0040336 (PMC2082648; doi:10.1371/journal.pmed.0040336)
Supplement: Figure S12 — The solid lines show the average incidence among unvaccinated people, the dashed lines among vaccinated people. (48 KB PPT) [file pmed.0040336.sg012.ppt]

## Slide 1
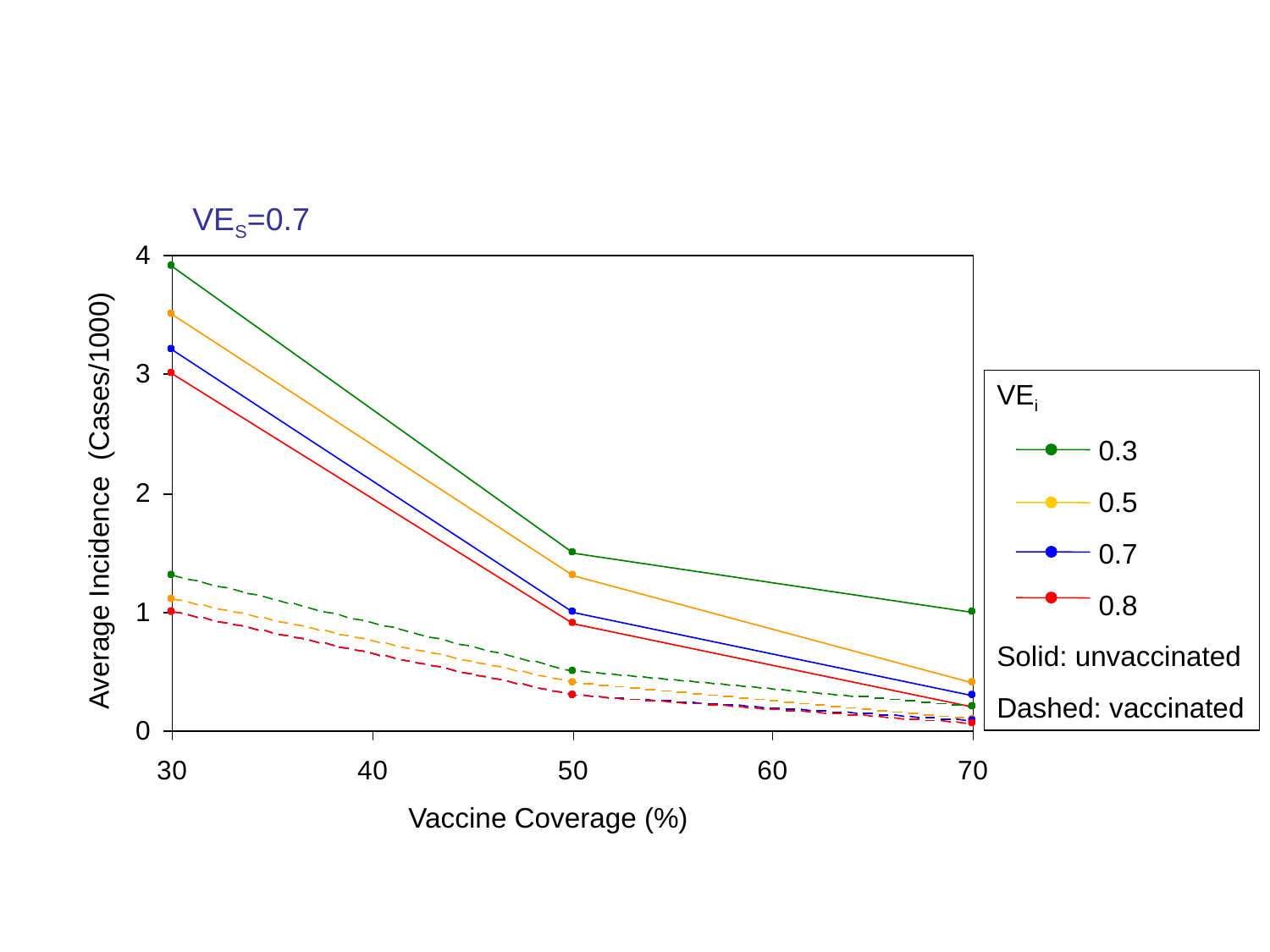

VES=0.7
VEi
 0.3
 0.5
 0.7
 0.8
Solid: unvaccinated
Dashed: vaccinated
 Average Incidence (Cases/1000)
Vaccine Coverage (%)
