# Supplementary material for: Controlling Endemic Cholera with Oral Vaccines
Source: PLoS Med. 2007 Nov 27;4(11):e336. doi: 10.1371/journal.pmed.0040336 (PMC2082648; doi:10.1371/journal.pmed.0040336)
Supplement: Figure S17 — (43 KB PPT) [file pmed.0040336.sg017.ppt]

## Slide 1
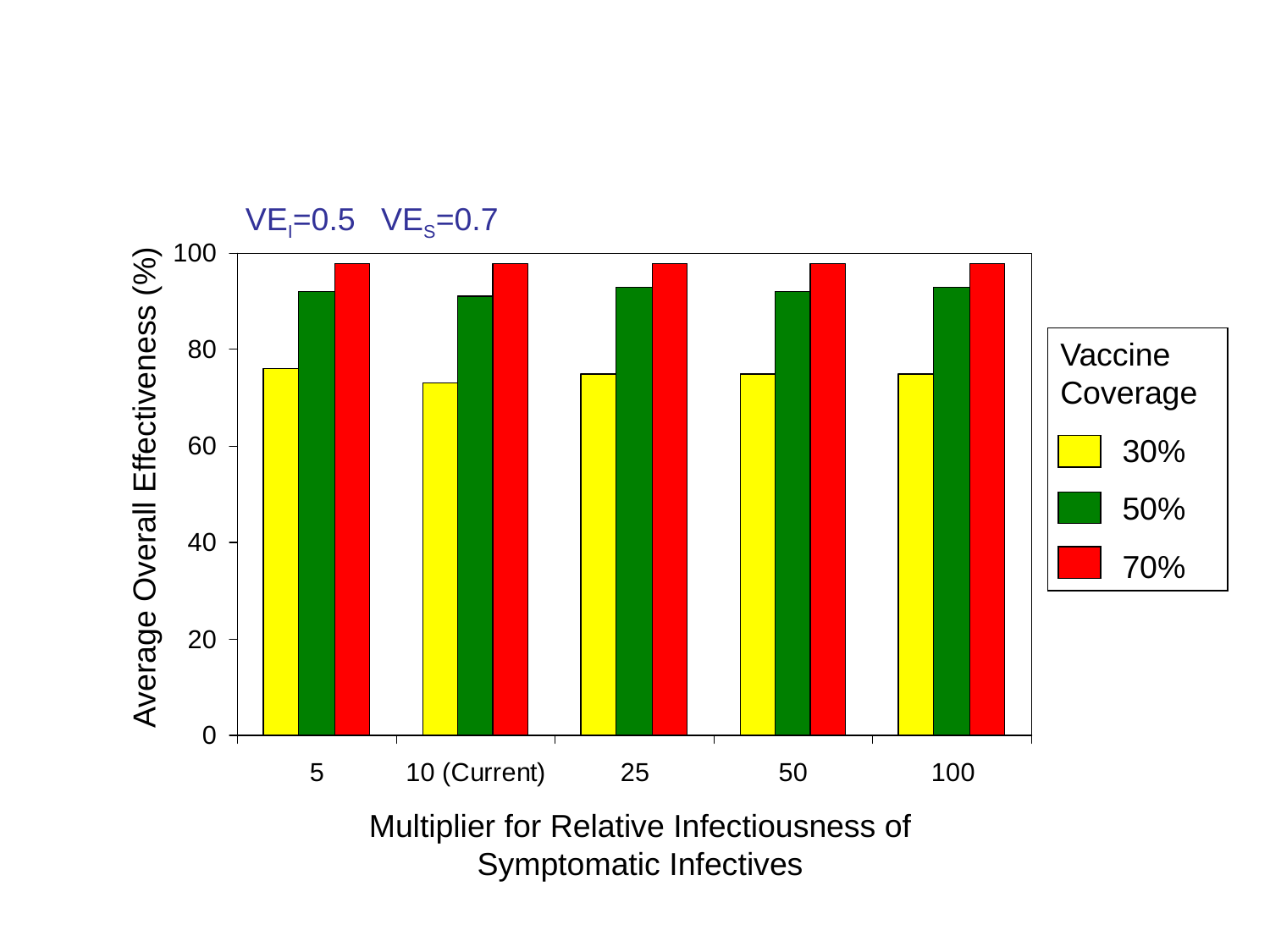

VEI=0.5	 VES=0.7
Vaccine Coverage
 30%
 50%
 70%
Average Overall Effectiveness (%)
Multiplier for Relative Infectiousness of Symptomatic Infectives
