# Supplementary material for: Controlling Endemic Cholera with Oral Vaccines
Source: PLoS Med. 2007 Nov 27;4(11):e336. doi: 10.1371/journal.pmed.0040336 (PMC2082648; doi:10.1371/journal.pmed.0040336)
Supplement: Table S1 — (33 KB DOC) [file pmed.0040336.st001.doc]

**Table S1**: Model Parameters and functions

| **Parameter** | **Description** | **Baseline value** |
| --- | --- | --- |
| $\pi$ | Transmission probability | 0.00000675 |
| R_0_ | Basic reproductive number | 5.0 |
| r | Relative susceptibility multiplier | 2 |
| b | Seasonal boost factor | 10 |
| VE_S_ | Vaccine efficacy for susceptibility | 0.7 |
| VE_I_ | Vaccine efficacy for infectiousness | 0.5 |
| * | Proportion infections symptomatic | 0.9 |
| * | Symptomatic relative infectiousness | 10 |
| * | Mean latent period | 3.6 days |
| * | Mean infectious period | 10.5 days |
| **Function** | **Description** |  |
| P(t) | Infection function for a person on day t | |
| u_i_ (t) | Number of unvaccinated infectious people in sub-region i on day t | |
| v_i_ (t) | Number of vaccinated infectious people in sub-region i, on day t | |

^*^No symbol is given in text.
