# Supplementary material for: Controlling Endemic Cholera with Oral Vaccines
Source: PLoS Med. 2007 Nov 27;4(11):e336. doi: 10.1371/journal.pmed.0040336 (PMC2082648; doi:10.1371/journal.pmed.0040336)
Supplement: Text S1 — (34 KB DOC) [file pmed.0040336.sd001.doc]

**Text File S1: Simulator Overview and Population Structure**

An overview of the simulator is shown in Figure S1. The stochastic simulator, coded in ANSI c, models cholera natural history (Fig. 1), daily infection transmission dynamics (based on an infection probability function) for a 180-day long cholera outbreak in Matlab, and the effect of mass vaccination strategies. The input population, described in detail in section 2 below, is a model of Matlab based on historical data [1]. Each run of the simulator is seeded with five, randomly chosen, unvaccinated, initial infectives.

The simulator tracks the infection status of each person in the population each day during each run, with cholera incidence rates in unvaccinated and vaccinated portions of the population being calculated at the end of the run. For each vaccination strategy investigated, 1000 runs of the simulator are performed and the cholera incidence rates and vaccine effectiveness (compared with runs where vaccine coverage is 0%) estimates averaged. The simulator produces the output necessary to produce videos (S1 and S2) of spatial-temporal epidemics at different vaccination coverage levels.

The model population for Matlab, Bangladesh, is based on the population of that area in 1985 as described by Ali, et al. [1]. The population of 183,826 matches, approximately, the age, sex, bari size and bari location distributions of the Matlab population in 1985 [1]. Baris are patrilineally related household clusters [1]. The age and bari size distributions are shown in Figures S2 and S3, respectively. Figure S4 A shows the location of Matlab in Bangladesh. Figure S4 B shows the Chandpur administrative district which contains Matlab. We mapped a rectangular grid onto the Matlab area and divided the grid into 64 similarly-sized sub-regions of about 6 square km in size (Figure S4 C). The study baris were contained within 43 of these sub-regions, shown in yellow in Figure S4 C. Figure S5 shows the spatial distribution of baris within this study area.

The model represents the number of contacts the typical person makes with sources of potential cholera transmission, mainly consisting of ponds and other water sources, each day. All people in the population are assumed to have exposure to sources of infection within their own sub-region. Fifty percent of males 14 to 16 years-old, and all males over 16 are assumed to work, possibly outside of their sub-region, where they can come into contact with additional sources of infection. For those men that work, we assigned each work sub-region was randomly assigned, as follows: 51% worked within their residence sub-region, 39% worked in a sub-region 4 to 10km away, and 10% worked in sub-regions more than 10km away from their residence sub-region. Distance-traveled-to-work data were not readily available for Matlab; we based this function on school travel time data reported in the Matlab Health and Socioeconomic Survey (MHSS) [2].

**References**

1. M Ali, M Emch, L von Seidlein, M Yunus, *et al.* Herd immunity conferred by killed oral cholera vaccines in Bangladesh: a reanalysis. *Lancet*, 366: 44-49, 2005.
2. O Rahman, J Menken, A Foster, P Gertler. MATLAB [BANGLADESH] HEALTH AND SOCIOECONOMIC SURVEY (MHSS), 1996 [Computer file]. 5th ICPSR version. Santa Monica, CA: RAND [producer], 2001. Ann Arbor, MI: Inter-university Consortium for Political and Social Research [distributor], 2001.
