# Supplementary material for: Controlling Endemic Cholera with Oral Vaccines
Source: PLoS Med. 2007 Nov 27;4(11):e336. doi: 10.1371/journal.pmed.0040336 (PMC2082648; doi:10.1371/journal.pmed.0040336)
Supplement: Text S2 — (45 KB DOC) [file pmed.0040336.sd002.doc]

**Text File S2: Infection Function**

The probability of infection for each susceptible individual residing or working in a particular sub-region, on each day, depends on several factors, including the vaccination status of the individual and the numbers of vaccinated and unvaccinated infectious people in the sub-region, and whether or not infectious people were symptomatic or asymptomatic. Another factor is the transmission probability, $\pi$, that an unvaccinated susceptible is infected when there is a single unvaccinated infectious person in the sub-region; this probability is determined by calibrating the simulator to historical incidence rate and vaccine effectiveness data.

If a susceptible individual is vaccinated, the transmission probability is multiplied by $\theta$, the relative susceptibility of a vaccinated person compared to an unvaccinated person; the vaccine efficacy for susceptibility is 1- $\theta$. For each vaccinated infectious person, the transmission probability is multiplied by $\varphi$, the relative infectiousness of a vaccinated person compared with an unvaccinated person. The vaccine efficacy for infectiousness is 1 – $\varphi$. The transmission probability is multiplied by the relative susceptibility multiplier, r, which is used to model the level of immunity in the population with respect to that of Matlab where cholera transmission has been active for many years. For example, if *r = 2,* then people in the population modeled are twice as susceptible to infection as those in Matlab*.*

The probability, *P(t)*, that a susceptible person is infected with cholera in sub-region *i* on day *t* is then given by:

$$P(t)=\left[1-(1-\theta^{x}br\pi)^{u_{i}(t)}(1-\theta^{x}\varphi br\pi)^{v_{i}(t)}\right]$$

Where

$\pi$ = probability that an unvaccinated susceptible is infected either from the

environment or from direct contact due to the presence of a single

unvaccinated infective in the sub-region (transmission probability),

*r =* relative susceptibility multiplier*.*

*x* = 1 if susceptible is vaccinated, 0 if unvaccinated,

$\theta$ = 1 – vaccine efficacy against susceptibility,

$\varphi$ = 1 – vaccine efficacy against infectiousness,

*u_i_ (t)* = number of unvaccinated infectious people in sub-region i on day t,

*v_i_ (t)* = number of vaccinated infectious people in sub-region i, on day t; and

*b* = seasonal boost factor for first 30 days of each run.

The vaccine efficacy against susceptibility is set to 0.7, and the vaccine efficacy against infectiousness to 0.5. The relative susceptibility multiplier, r, is set to 1 to model Matlab, or to higher values to model populations which have less immunity than Matlab. For example, if

r = 2, then people in the population being modeled are twice as susceptible to infection as those in Matlab. The seasonal boost factor is set to 10. In order to calibrate the model to the incidence rate and direct effectiveness data in Ali, et al. [1], the transmission probability, $\pi$, was varied until the difference between the observed and simulated cholera incidence rates was minimized. This was achieved at the value $\pi$ = 0.00000675. This corresponds to a reproductive number of 5.0 with a standard deviation of 3.3, after the seasonal boost occurs.

We define the reproductive number as the average number of people that a typical infected person infects both directly and through environmental sources over the course of their infectious period at the time that the seasonal boost occurs. This reproductive number is for the level of natural immunity that is present in the population at the time that the seasonal boost occurs. In order to calculate the reproductive number from our model, for a given value of $\pi$, we randomly select a typical initial infective from the population. We then insert this person into the population where everyone else's ability to transmit is 0. We then count the number of secondary infections. This is repeated 1000 times. In this way, we generate the whole distribution of secondary cases due to a typical infected person.

*P(t)* is evaluated for each susceptible individual, on each day of each run of the simulator. A Bernoulli trial is conducted by generating a uniform [0, 1] random number. If the number is less than *P(t)*, the susceptible individual becomes infected and enters the latent phase of infection (Figure 1). Thus, the infectious transmission process is a nonlinear function of the number of susceptible and infectious people each day.
