# Supplementary material for: Controlling Endemic Cholera with Oral Vaccines
Source: PLoS Med. 2007 Nov 27;4(11):e336. doi: 10.1371/journal.pmed.0040336 (PMC2082648; doi:10.1371/journal.pmed.0040336)
Supplement: Text S3 — (34 KB DOC) [file pmed.0040336.sd003.doc]

**Text File S3: Model Calibration and Sensitivity Analyses**

**Model Calibration:** The simulator was calibrated to the cholera incidence and vaccine direct effectiveness data reported by Ali et al. [1], shown in Table 1. Figure 1 shows the epidemic curves and average incidence rates for calibration runs which were performed with 0%, 14%, 38% and 58% vaccination coverage among children 2-15 years of age and women. A summary of baseline parameter values and dynamic functions is given in Table S1.

**Mass Vaccination Strategies:** Figure S6 shows typical epidemic curves and average incidence rates for runs performed with 0%, 30%, 50% and 70% vaccination coverage in the entire population (two years and older in age). The average cholera incidence rates for scenarios with 30% to 70% vaccine coverage are given in Figure S7. The corresponding average vaccine effectiveness is given in Table 2. Figures S6 and S7 show that the average incidence rate is less than two cases per thousand when vaccination coverage is 50% or higher.

**Sensitivity Analyses for VE_S_ and VE_I_:** Sensitivity analyses for values of the vaccine efficacy against susceptibility (VE_S_) and the vaccine efficacy against infectiousness (VE_I_) ranging from 0.3 to 0.8 were conducted. Figure S8 shows that, for VE_I_=0.5 and VE_S_ set to a low value of 0.3, vaccination coverage of at least 60% in the entire population (two years and older in age) would be needed to reduce average incidence rates to below two cases per thousand; however, with a VE_S_ of 0.5 or higher, 50% coverage would be adequate. Figures S9, S10 and S11 show that the indirect, total and overall vaccine effectiveness exceed 80% when the vaccination coverage level is at least 50%.

Figure S12 shows that, even for VE_S_=0.7 and VE_I_ set to a low value of 0.3, the average incidence rates would be below four cases per thousand at a vaccination coverage of 30% and below two cases per thousand with vaccination coverage of 50% or higher. Figures S13, S14 and S15 show that the indirect, total and overall vaccine effectiveness exceed 80% when the vaccination coverage level is at least 50%.

**Additional Sensitivity Analyses:** Sensitivity analyses were performed for values of the seasonal boost factor, the relative infectiousness of symptomatic infectives, and for varied sub-region sizes. Figure S16 shows that, with vaccination coverage of at least 50%, the average overall vaccine effectiveness exceeds 80% for values of the seasonal boost factor ranging from 2 to 50. Figure S17 shows that, with vaccination coverage of at least 50%, the average overall vaccine effectiveness exceeds 80% for values of the multiplier for the relative infectiousness of symptomatic infectives from 5 to 100. Figure S18 shows that, with vaccination coverage of at least 50%, the average overall vaccine effectiveness when larger sub-regions (> 6km^2^ each) are modeled is at least 80%. For very small sub-regions (0.04 km^2^ each), the average overall vaccine effectiveness approaches 75% with a vaccination coverage of 70%.
